# The mRNA vaccine BNT162b2 demonstrates impaired T_H_1 immunogenicity in human elders *in vitro* and aged mice *in vivo*

**DOI:** 10.21203/rs.3.rs-2395118/v1

**Published:** 2022-12-21

**Authors:** Byron Brook, Benoit Fatou, Abhinav Kumar Checkervarty, Soumik Barman, Cali Sweitzer, Anna-Nicole Bosco, Amy C. Sherman, Lindsey R. Baden, Elena Morrocchi, Guzman Sanchez-Schmitz, Paolo Palma, Etsuro Nanishi, Timothy R. O’Meara, Marisa E. McGrath, Matthew B. Frieman, Dheeraj Soni, Simon D. van Haren, Al Ozonoff, Joann Diray-Arce, Hanno Steen, David J. Dowling, Ofer Levy

**Affiliations:** 1 *Precision Vaccines Program*, Division of Infectious Diseases, Boston Children’s Hospital, Boston, MA, USA.; 2 Department of Pediatrics, Harvard Medical School, Boston, MA, USA.; 3 Department of Pathology, Boston Children’s Hospital, Harvard Medical School, Boston, MA, USA.; 4 Prevention of Organ Failure (PROOF) Centre of Excellence, St Paul’s Hospital, University of British Columbia, Vancouver, BC, Canada.; 5 UBC Centre for Heart Lung Innovation, Providence Research, St Paul’s Hospital, Vancouver, BC, Canada.; 6 Department of Medicine, Brigham and Women’s Hospital, Boston, MA, USA.; 7 Academic Department of Pediatrics (DPUO), Research Unit of Clinical Immunology and Vaccinology, Bambino Gesù Children’s Hospital, IRCCS, Rome, Italy.; 8 Bambino Gesù Children’s Hospital, Rome, Italy.; 9 Chair of Pediatrics, University of Rome, Tor Vergata, Italy.; 10 Center for Pathogen Research, Department of Microbiology and Immunology, The University of Maryland School of Medicine, Baltimore, MD, USA.; 11 Broad Institute of MIT & Harvard, Cambridge, MA, USA.

## Abstract

mRNA vaccines have been key to addressing the SARS-CoV-2 pandemic but have impaired immunogenicity and durability in vulnerable older populations. We evaluated the mRNA vaccine BNT162b2 in human *in vitro* whole blood assays with supernatants from adult (18–50 years) and elder (≥60 years) participants measured by mass spectrometry and proximity extension assay proteomics. BNT162b2 induced increased expression of soluble proteins in adult blood (e.g., C1S, PSMC6, CPN1), but demonstrated reduced proteins in elder blood (e.g., TPM4, APOF, APOC2, CPN1, and PI16), including 30–85% lower induction of T_H_1-polarizing cytokines and chemokines (e.g., IFNγ, and CXCL10). Elder T_H_1 impairment was validated in mice *in vivo* and associated with impaired humoral and cellular immunogenicity. Our study demonstrates the utility of a human *in vitro* platform to model age-specific mRNA vaccine activity, highlights impaired T_H_1 immunogenicity in older adults, and provides rationale for developing enhanced mRNA vaccines with greater immunogenicity in vulnerable populations.

## Introduction:

Severe acute respiratory syndrome coronavirus 2 (SARS-CoV-2) mRNA vaccines were developed and rapidly implemented to address the public health threat of coronavirus disease 2019 (COVID-19). The originally observed ~90–95% efficacy of BNT162b2 (Comirnaty) and mRNA-1273 (Spikevax) against wildtype SARS-CoV-2, reducing severe disease and/or hospitalization^1,2^, surpassed previous immunization efforts for other respiratory infections like influenza (with yearly efficacy between 10–80%^3,4^). The self-adjuvantation effects, particularly from ionizable lipids and mRNA^5–8^, can enhance immunogenicity. Self-adjuvantation has been associated with high efficacy in live-attenuated vaccines, arising from pathogen-associated molecular patterns activating and enhancing innate immune responses^9–11^. Investigating mRNA vaccine-induced immune activation may provide insight into human- and age-specific immunogenicity.

Advancing age has been associated with impaired functionality including reduced neutralization, phagocytosis, chemotaxis, and co-stimulatory molecule expression^12,13^. These effects can contribute to lower T follicular helper CD4^+^ T cell (T_FH_) activity and B cell class switching, and increased T regulatory cell (T_reg_) frequency restraining responses^12–17^. Likely reflecting such immunosenescence, SARS-CoV-2 mRNA vaccines have been less effective at preventing hospitalization and symptomatic infection with a 9.5% impairment in older populations (>65 years, Y) versus adults (18–65Y)^2^, and 20% less efficacy in >80Y compared to 60–69Y^18^. Vaccine effectiveness (VE) has also been impacted, with a meta-analysis identifying a 9.3% decrease of VE preventing infection in older populations compared to the general populace^19^, and another review identifying consistently lower VE in those ≥65 than <65, with up to 15.3% less VE in the elderly^20^. Generally, age-associated susceptibility has been attributed to increased disease severity and reduced vaccine-induced protection^18,21–23^. Vulnerability, linked with immunosenescence in older adults, has been observed both with respect to SARS-CoV-2 infection induced immunogenicity^24^ and reduced immunogenicity of mRNA vaccines^12,25–29^. These observations mirror impaired immunogenicity responses in elders across a range of vaccines targeting encephalitis viruses, tetanus toxin, *Salmonella spp.*, *Streptococcus pneumoniae*, influenza, and varicella zoster virus^13–17^, impacting both humoral and cell-mediated immunity, culminating in diminished VE in the elderly^26,30–32^.

Age-associated T-cell changes could contribute to reduced cellular and antibody (Ab) functionality following mRNA vaccination of aged groups^25–27^. Older populations (>60Y, >65Y, and >80Y) have had lower cell mediated immunity (CMI), with impaired CD4^+^ and CD8^+^ activation following BNT162b2 immunization, compared to adults^12,29,33^. Impaired CD4^+^ and CD8^+^ T cell responses were also observed in older adults post-SARS-CoV-2 infection^34^, suggesting consistent age-dependent immunological differences. Additional immunizations with mRNA vaccines encoding Wuhan1 or bivalent Wuhan1 with BA.4/BA.5 mRNA encoding spike protein have been applied to overcome elder immunosenescence^35–37^. The third immunization with Wuhan1 mRNA had amplified immunogenicity against Wuhan1, and the variants Delta (B.1.617.2), and Omicron (B.1.1.529) transiently^35^, but failed to induce durable immunity as >65Y individuals had more rapid waning of immunity compared to <65Y^36^. The emergency use authorization of a 4^th^ immunization in elders^37^ and bivalent mRNA vaccination lack the formulation changes needed to increase elder responsivity. We hypothesized that elder participant samples stimulated with BNT162b2 would have innate immune impairments that could contribute to the observed impaired adaptive immune induction observed in the elderly. This has been broadly observed against other pathogens and immune stimuli^38^.

Modeling human immune activation *in vitro* employing primary human leukocytes and autologous plasma is a powerful tool to characterize vaccines, providing species- and age-specificity, and enabling paired comparisons of multiple conditions from the same study participant^22,39,40^. We hypothesized that elder participants could have differential immune activation in response to BNT162b2 stimulation, skewing the immune response towards a distinct activation state compared to adults, particularly an impaired production of analytes polarizing towards CD4^+^ T helper cell (T_H_) 1 differentiation. T_H_1 polarized immune responses can trigger effective intracellular pathogen responses^41^, including CD8^+^ T cell-mediated immunity^42^, alongside B cell class switching^43,44^ and induction of T_FH_-like activity for effective B cell responses in the absence of T_FH_^45^, functions that have been impaired in mRNA immunized elder individuals. To gain further insight into age-dependent mRNA vaccine responses, we characterized BNT162b2 immune stimulation in supernatants from adult and elder human whole blood assay (WBA) *in vitro* using targeted high performance liquid chromatography, mass spectrometry (LC/MS) proteomics, a second proteomics platform of high sensitivity dual Ab-based proximity extension assay (PEA; “*Olink”*), and a multiplex quantifying cytokines and chemokines. We also verified T_H_1 responses of age dependent BNT162b2 induced immunogenicity in young and aged mice, *in vivo*. We identified impaired T_H_1 polarization in both humans and mice that may contribute to the lower mRNA vaccine-induced humoral and cellular immunogenicity observed in elders.

## Results:

Human study participants donated peripheral blood which was evaluated *in vitro* for immune activation following stimulation with the BNT162b2 lipid nanoparticle (LNP) encapsulated mRNA vaccine (*Pfizer/BioNTech*). Participants were grouped by age, with younger and middle-aged adults 18–50Y, and elderly, older adults ≥60Y (Table S1).

**Fig. 1:**
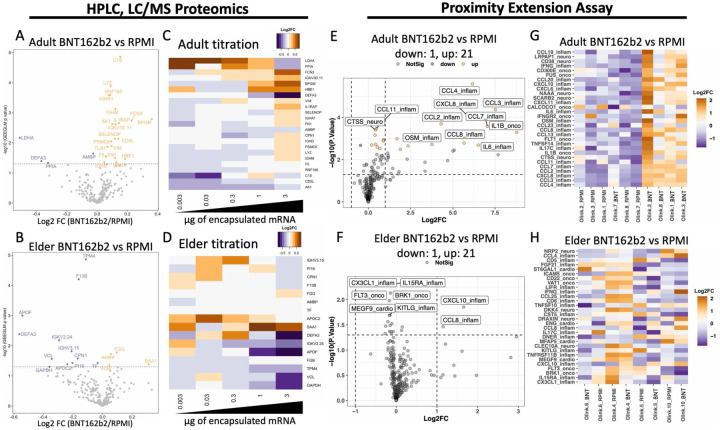
SARS-CoV-2 BNT162b2 mRNA vaccine induced robust inflammatory protein profile in supernatants of adult but not elder human whole blood *in vitro*. Adult (18–50 years, Y) and elder (≥65Y) whole blood was stimulated *in vitro* with vehicle (RPMI) or BNT162b2 (BNT) vaccine containing mRNA encapsulated in lipid nanoparticle. Supernatants were characterized by mass spectrometry proteomics (**A-D**) and Proximity Extension Assay (PEA, **E-H**). Volcano plots of linear modelling identified significantly down- (purple) and up-regulated (orange) protein expressions presented with log_2_ fold change (FC) from (**A**) adult and (**B**) elder participant samples. A supervised heatmap of average log_2_FC per BNT162b2 dose induced differentially expressed proteins (DEPs) was evaluated with noted (**C**) up-regulation of proteins in adult and (**D**) down-regulation of proteins in elder samples stimulated with 0.003μg to 3μg of mRNA within BNT162b2. PEA evaluation of samples was presented as volcano plots with log_2_FC of 3 μg mRNA within BNT162b2-stimulations over matching vehicle control from (**E**) adult and (**F**) elder participant samples. Significant BNT162b2-induced down-regulated or up-regulated proteins following BNT162b2 stimulation, with the top 10 DEP labelled, alongside a dashed horizontal grey line denoting a nominal p-value of 0.05. PEA-quantified proteins were color-coded following p-value adjustment, with purple down-regulated, orange up-regulated, and grey not significant. An unsupervised heatmap of the top 30 significant DEPs from comparing BNT162b2 (LNP) to vehicle control (RPMI) in (**G**) adult and (**H**) elder participant samples had 4/5 adult samples cluster while elders were more heterogeneous. Proteins were listed with the associated Target 96 platform (Inflammation, ‘inflam’; Oncology, ‘onco’; Neurology, ‘neuro’; Cardiology III, ‘cardio’). Sample sizes were (A-D) n = 12–14 and (E-H) n = 4–5. Significance was evaluated by (A-D) GEEGLM analysis with nominal p-value < 0.05, and (E-H) moderated T-test reporting (E) adjusted and (F) nominal p-values < 0.05. (G-H) Euclidean-clustered heatmaps from individual donors’ (‘Olink_#’) vehicle control (‘RPMI’) and BNT162b2 stimulated conditions (‘LNP’).

Heparinized human whole blood was stimulated with BNT162b2 in a whole blood assay (WBA) to measure both immune activation and age-associated differences by supernatant proteomics. LC/MS has been employed to identify disease severity-associated responses after SARS-CoV-2 infection in humans^46^, and could provide insight into immune activation from BNT162b2 stimulation. Protein expression was evaluated by generalized estimating equations generalized linear model (GEEGLM) analysis^47–49^, assessing fold change (FC) over matching vehicle control for baseline-normalization. Adult participants had 20 upregulated and 4 downregulated proteins (Fig. 1A), while elder participants demonstrated 4 upregulated and 12 downregulated proteins (Fig. 1B). Heatmap visualization displayed dose- and age-dependent up- and down-regulation, respectively (Fig. 1C–D). BNT162b2-induced proteins in adults included complement component 1s (C1S), a proteasome regulatory unit protein (PSMC6), hemoglobin subunit epsilon (HBE1), and carboxypeptidase N catalytic chain (CPN1). Downregulated proteins included peptidyl-prolyl *cis-trans* isomerase A (PPIA) and neutrophil defensin 3 (DEFA3). Elder sample protein upregulation included serum amyloid A-1 protein (SAA1) and the fibrinogens-γ and -β (FGG and FGB), while downregulated proteins included DEFA3, tropomyosin alpha-4 chain (TPM4), apolipoprotein F (APOF), apolipoprotein C-II (APOC2), CPN1, and peptidase inhibitor 16 (PI16). Additional supernatant evaluation was performed with 4 Target 96 kits (inflammation, inflam; oncology III, onco; neurology, neuro; and cardiometabolic, cardio) of PEA-based proteomics quantifying 368 proteins. PEA assessment identified upregulation of predominantly inflammatory markers in adult BNT162b2-stimulated samples compared to vehicle (e.g., chemokine (C-C motif) ligand 2 (CCL2), CCL3, CCL4, CCL7, CCL8, CCL11, chemokine (C-X-C motif) ligand 8 (CXCL8), IL-1β, and IL-6^46,50–56^, Fig. 1E). Only CCL8 and CXCL10 were induced in BNT162b2-stimulated elder samples, compared to vehicle control (Fig. 1F). Unsupervised heatmap visualization demonstrated 4/5 adult samples clustered by treatment versus no clustering in the elder study participants (Fig. 1G–H).

**Fig. 2:**
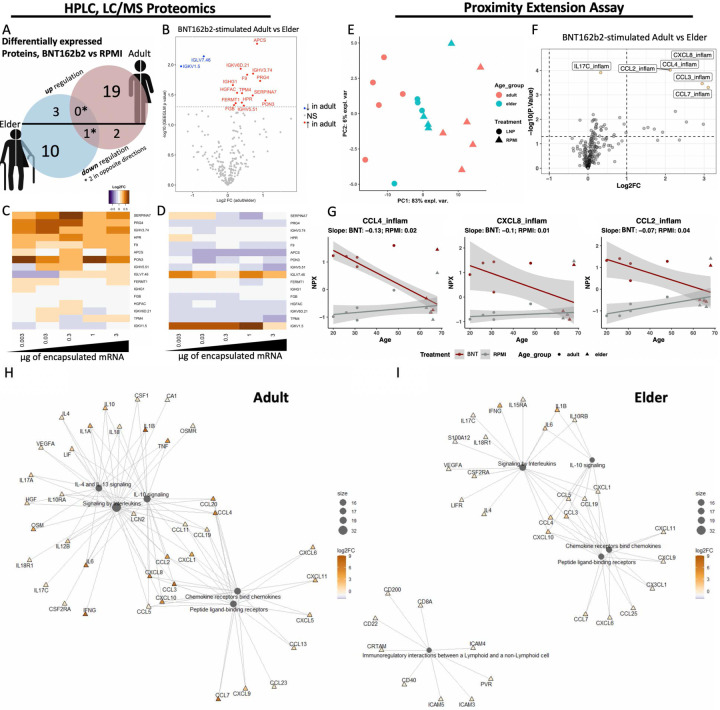
Lower BNT162b2-induced inflammatory response in elder vs adult whole blood assayed *in vitro*. Whole blood assay supernatant samples, as described in Fig. 1, were evaluated by mass spectrometry-based proteomics (A-D) and PEA-based protein quantification (E-I). (**A**) Up-/down-regulated DEPs from BNT162b2-stimulated samples against paired vehicle controls yielded predominantly distinct age group patterns. (**B**) Comparing stimulated samples from elders and adults yielded 16 significant proteins (p < 0.05), 14 of which had a negative log_2_ fold change (FC) compared to adult, with a horizontal line denoting nominal p value (p = 0.05). A supervised heat map of these significant age-comparison proteins highlight (**C**) adult up-, and (**D**) elder downregulation. Additional PEA-evaluated supernatant proteomics assessed age-specific differences between 3 μg mRNA within BNT162b2-stimulated adult and elder samples. (**E**) Principal component analysis (PCA) visualization of PEA-quantified proteins displayed separation between BNT162b2-stimulation (circles) and RPMI (triangles), but only in adults (red). Elders (teal) displayed mostly overlapping responses between treatments. (**F**) Volcano plot analysis identified multiple inflammatory markers with greater upregulation in adults than in elders with a horizontal line denoting nominal p value < 0.05, and colored points denoting those adjusted p value < 0.05. (**G**) Normalized protein expression (NPX) graphed against age (Y) at sampling identified significantly negative correlation of BNT162b2-induced CCL4 (p = 0.04). The top 100 PEA-identified DEPs were converted to gene names and evaluated by enrichment analysis, demonstrating mRNA vaccine-induced network changes in (**H**) adults and (**I**) elders. Nodes were color-coded by degree of induction and sized by number of contributing proteins. Sample sizes were (A-D) n = 12–14 and (E-I) n = 4–5. Significantly induced proteins were evaluated by GEEGLM analysis, determined to be significant with (A, B) nominal and (F) moderated T-test with adjusted p-values < 0.05. (G) Significant age effects were evaluated by Pearson’s correlation with nominal p-values.

BNT162b2-induced proteomic protein profiles in the WBA differed by age, with adults and elders expressing 21 and 13 unique proteins, respectively (Fig. 1A–B, 2A). Only 3 significant differentially expressed proteins (DEPs) overlapped across age groups, with only 1, DEFA3, downregulated in both age groups. The other 2 proteins had different directionality (Alpha-1 microglycoprotein, AMBP, was downregulated in adult but upregulated in elder, and vice versa for CPN1). BNT162b2 generally induced greater responses in adults vs. elders (Fig. 2B–D). Principal component analysis (PCA) clustering of PEA proteomics displayed distinct adult and elder patterns with separation only in adult BNT162b2-stimulated vs Roswell Park Memorial Institute Medium (RPMI) control (Fig. 2E). PEA comparison of just BNT162b2-stimulated adult and elder samples also demonstrated a generally greater upregulation of inflammatory markers in adults (Fig. 2F). Advancing age had a negative correlation for BNT162b2-induced CCL4 (BNT162b2 stimulation slope −0.13, p = 0.04), and trends towards lower CXCL8 (BNT162b2 stimulation slope −0.1, p = 0.09), and CCL2 expression (BNT162b2 stimulation slope −0.07, p = 0.11) (Fig. 2G). Network representation of DEP pathway analyses indicated some similar pathways induced in adult and elder participant samples (Fig. 2H–I, e.g., “signaling by interleukins”). Elder sample profiles had fewer proteins contributing to each pathway node and an additional predominantly down regulated “immunoregulatory interactions between a lymphoid and a non-lymphoid cell” node that was not observed in adult study participants. Additionally, the “IL-4 and IL-13 signaling” enriched in adult samples were not observed in elder samples. Overall, evaluation of human *in vitro* BNT162b2 stimulation identified broadly dampened immune responses in elder participant samples across 2 proteomic platforms.

**Extended Data Fig. 1:**
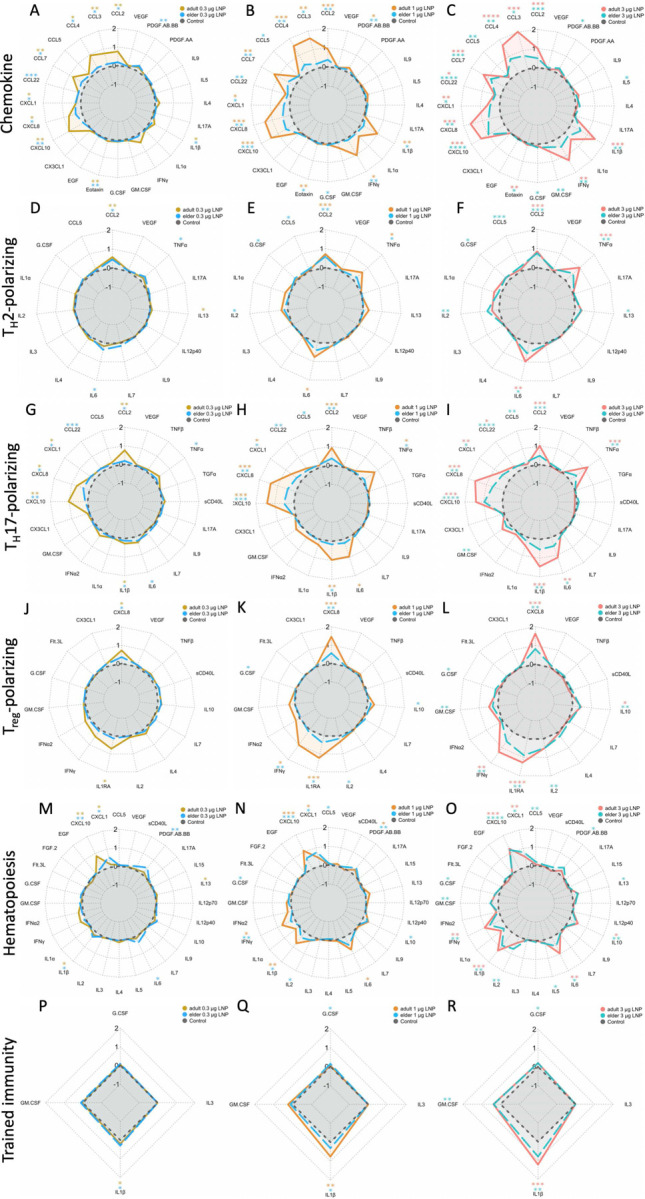
mRNA vaccine induces concentration-dependent analyte induction. Data from 41-plex cytokine and chemokine analysis of WBA supernatants were analyzed for (**A-C**) chemokine, (**D-F**) T_H_2-polarizing, (**G-I**) T_H_17-polarizing, (**J-L**) T_reg_-polarizing, (**M-O**) hematopoiesis-supporting, and (**P-R**) secondary vaccine effects (trained immunity, innate memory, nonspecific effects) based on functionally associated analytes as summarized in Supplementary Table 2. Each radar plot displays on the vertical spoke the Log_10_-transformation of the fold change of analyte production divided by negative control (black) evaluated in adult (orange-red lines) and elder (blue-teal lines) WBA samples from escalating BNT162b2 mRNA weights of 0.3 μg (left, A, D, G, J, M, P), 1 μg (middle, B, E, H, K, N, Q), and 3 μg (right, C, F, I, L, O, R). Significance presented above each analyte was from one-sided unpaired T-tests comparing BNT162b2 stimulated to negative control, color-coded by age group (red adult, teal elder). Significance across function was evaluated by GEEGLM analysis testing for age-dependent differences by evaluating age-interaction with function. Elder versus adult 95% confidence intervals (CI) calculated by adding or subtracting 1.96 times standard error were (A-C) 0.86–1.11, (D-F) 0.94–1.15, (G-I) 0.97–1.08, (J-L) 0.94–1.13, (M-O) 0.93–1.41, and (P-R) 0.99–1.12 for each analyte function. Sample sizes were (A-C) n = 8–9, (D-F) n = 8–9, (G-I) n = 5–8, (J-L) n = 4–7, (M-O) n = 7–9, and (P-R) n = 12–14, differing across ages and due to excluded samples with missingness across any of the measured analytes or amounts of BNT162b2 stimulation. Significance was denoted with * p < 0.05, ** p < 0.01, *** p < 0.001, **** p < 0.0001.

**Fig. 3:**
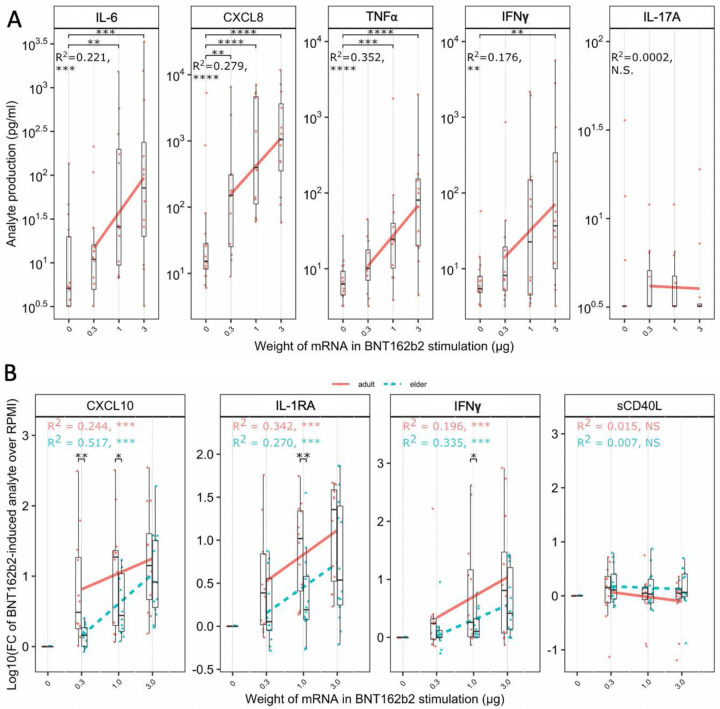
BNT162b2 induced concentration- and age-dependent cytokine and chemokine production in human whole blood stimulated *in vitro*. Adult and elder whole blood samples were stimulated for 24 hr with RPMI control (0 μg), or with 0.3, 1, or 3 μg of mRNA within the BNT162b2 vaccine. Supernatant was assessed for induction of 41 cytokines and chemokines by multiplex assay. (**A**) Significant concentration-dependent induction of IL-6, CXCL8, TNFα, and IFNγ was noted in adults, while other analytes like IL-17A was not. A red best-fit linear modelling (LM) line of analyte production in stimulated conditions following log_10_-transformation overlays the boxplots, indicating dose dependency for each except IL-17A. (**B**) Adult participant samples (solid red line) were compared to elder participant samples (dashed blue line), with baseline standardization of log_10_ transformed Fold Change (FC) of stimulated over matching vehicle (RPMI) control. Presentation of CXCL10, IL.1RA, and IFNγ had age-dependent differences while sCD40L was not induced. (A) Shapiro-Wilk then Wilcoxon rank-sum tests evaluated matched analyses of stimulated versus vehicle control. LM was evaluated with log_10_-transformation-normalization of cytokine production with R^2^ and significance annotated by age. (B) Age group comparisons were evaluated by 1-sided unpaired T-tests, and significant dose-dependent cytokine induction was evaluated by LM comparing log_10_-transformed fold change of 0.3, 1, and 3 μg mRNA within BNT162b2-stimulated whole blood over vehicle control. Boxplots display median with interquartile range and significance is denoted by * p < 0.05, ** p < 0.01, *** p < 0.001, **** p < 0.0001 with n = 12–14.

**Fig. 4:**
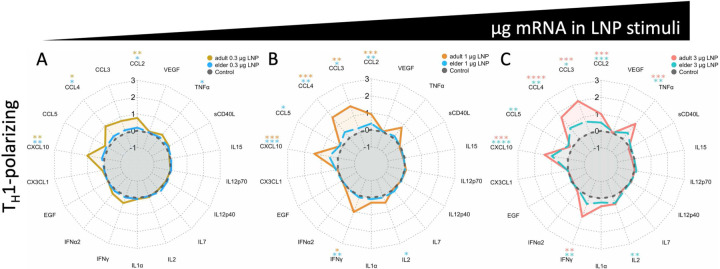
Impaired BNT162b2-induced T_H_1 polarizing cytokine production in human elder blood. Multiplex-quantified analytes were categorized as T_H_1 polarizing based on previously described functions summarized in Table S2. Each radar plot displays the per-group average of Log_10_-transformation of the fold change of analyte production divided by vehicle (RPMI) control (black) per spoke, in adult (orange-red lines) and elder (blue-teal lines) WBA stimulated with mRNA (encapsulated in BNT162b2) weights of (A) 0.3 μg, (B) 1 μg, and (C) 3 μg. Significance presented above each analyte displays one-sided unpaired T-tests compared to vehicle control, with color-coded asterixis (orange adult, teal elder). GEEGLM analyses evaluating age-dependent functional differences demonstrated 7.4% less T_H_1 cytokine production in elder participant samples vs adult samples (p = 0.027). Percent difference was calculated by exponentiating the point-estimate. Sample sizes for A-C were n = 5–8, excluding samples with missingness. Significance is denoted by * p < 0.05, ** p < 0.01, *** p < 0.001, **** p < 0.0001.

An additional evaluation by a targeted multiplex bead-based assay identified titratable production of interleukin-6 (IL-6), CXCL8, tumor necrosis factor alpha (TNFα), and interferon gamma (IFNγ) in adult WBA samples (Fig. 3A). Other cytokines such as IL-17A were not induced. Adult and elder responses were fold change-normalized with stimulated over paired RPMI vehicle control (Fig. 3B). Evaluation of fold-induction of mRNA-stimulated over RPMI control identified induction of multiple cytokines in both age groups, including CXCL10, IL-1RA, and IFNγ. Nevertheless, across multiple stimulation doses, elder samples had 30–59% lower IFNγ, 42–85% lower CXCL10, and 54–85% lower IL-1RA FC induction compared to adults. Multiplex-quantified analytes were grouped by function (per Table S2) as T_H_1, T_H_2, T_H_17, or T regulatory (T_reg_) polarizing, chemokine, hematopoiesis-supporting, and trained immunity-associated. A linear modelling analysis, GEEGLM, evaluated if age interacted with each function. T_H_1 support was significantly impaired (p = 0.027) in elders compared to adults, by an average of 7.4% less in each analyte involved (Fig. 4A–C). The other functions evaluated were not significantly different (Extended data Fig. 1) indicating a predominant impairment in inducing analytes capable of inducing T_H_1 polarization.

**Extended Data Fig. 2:**
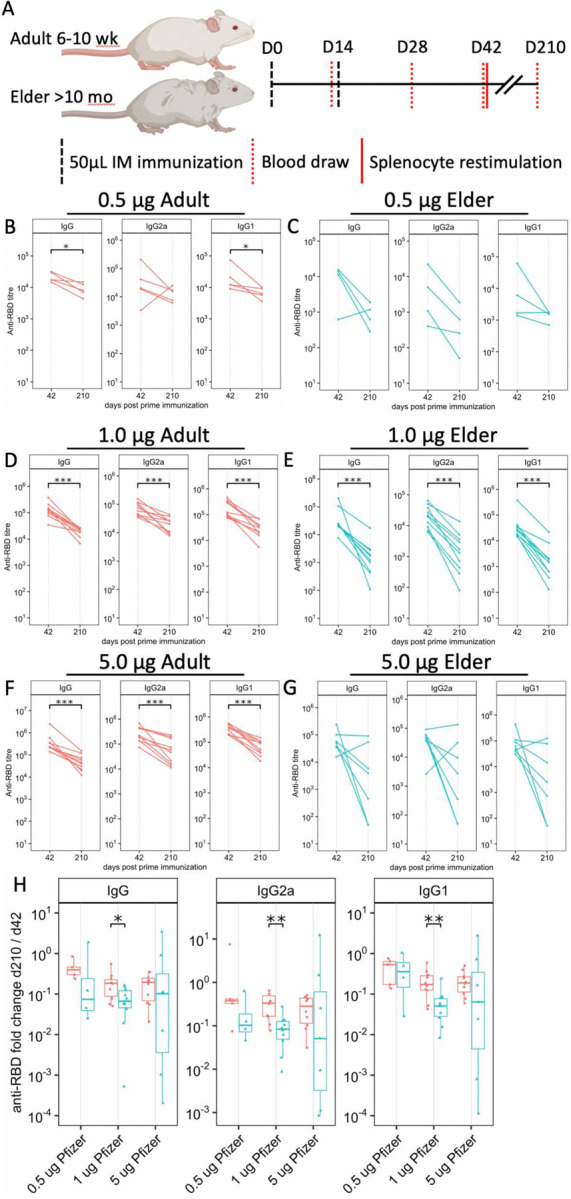
A murine model demonstrating greater waning of immunity in elder mice. (**A**) Murine immunizations were 50 μL injectant administered intramuscularly (IM, vertical dashed black line) following a prime (Day 0, D0), boost schedule, separated by 14 days. Retroorbital blood draws (vertical dotted red line) were collected pre-boost (D14), and D28, D42, and D210 post-prime immunization, with splenocytes (solid vertical red line) isolated on D42–46. Adult (6–12 weeks old, red) and Elder (>10 months old, blue) mice were immunized by intramuscular (IM) injection with 0.5 (**B-C**), 1 (**D-E**), or 5 (**F-G**) μg of mRNA in BNT162b2). Day 42 and 210 post-prime sera were collected for evaluation of binding capacity to RBD quantifying total IgG (left), IgG2a (middle), and IgG1 (right). (**H**) Waning immunity was evaluated by dividing D210 by the paired D42 for each age group and Ab isotype to evaluate relative differences between adult and elder mice. (B-H) Sample sizes: N = 4–10. Shapiro-Wilk test for normality B-H was followed by (B-H) paired 2-sided Wilcoxon rank-sum test comparing D210 vs D42, and (H) unpaired 1-sided Wilcoxon rank-sum test comparing age groups.

**Extended Data Fig. 3:**
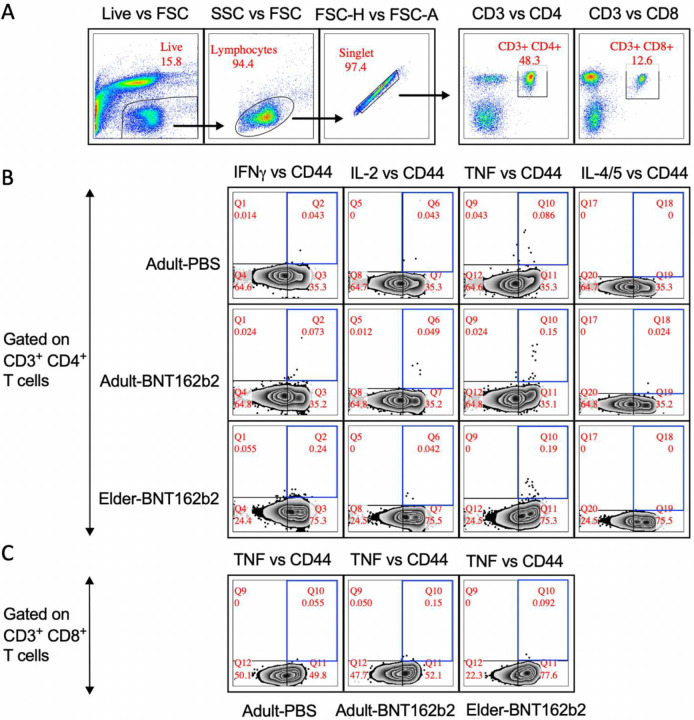
Flow cytometry gating strategy to characterize murine splenocyte spike specific CD4^+^ and CD8^+^ T cells. Murine splenocytes were collected on D42 post-prime, D28 post-boost. Spike (wild-type) antigen-specific T cell responses were evaluated by peptide stimulation and defined as CD4+/CD44+/cytokine+. (**A**) Shown is an example of the hierarchical gating strategy identifying live, singlet, CD3^+^CD4^+^ or CD3^+^CD8^+^, CD44^+^ and cytokine^+^ T cells. The first category listed, corresponds to y-axis (e.g., “Live”), while the second listed represents the x-axis (e.g., forward side scatter, FSC). The second panel also evaluated side scatter (SSC) against FSC to identify lymphocytes while the third panel evaluated both height (FSC-H) and area (FSC-A) of FSC for doublet exclusion. (**B**) The three rows denote representative CD3^+^CD4^+^ T cells from splenocytes of adult mice immunized with PBS (top), adult mice immunized with BNT162b2 (middle), or elder mice immunized with BNT162b2 (bottom). (**C**) CD3^+^ CD8^+^ T cells were evaluated with representative panels of adult mice immunized with PBS (left) or BNT162b2 (middle), or elders immunized with BNT162b2 (right) displayed.

**Figure 5:**
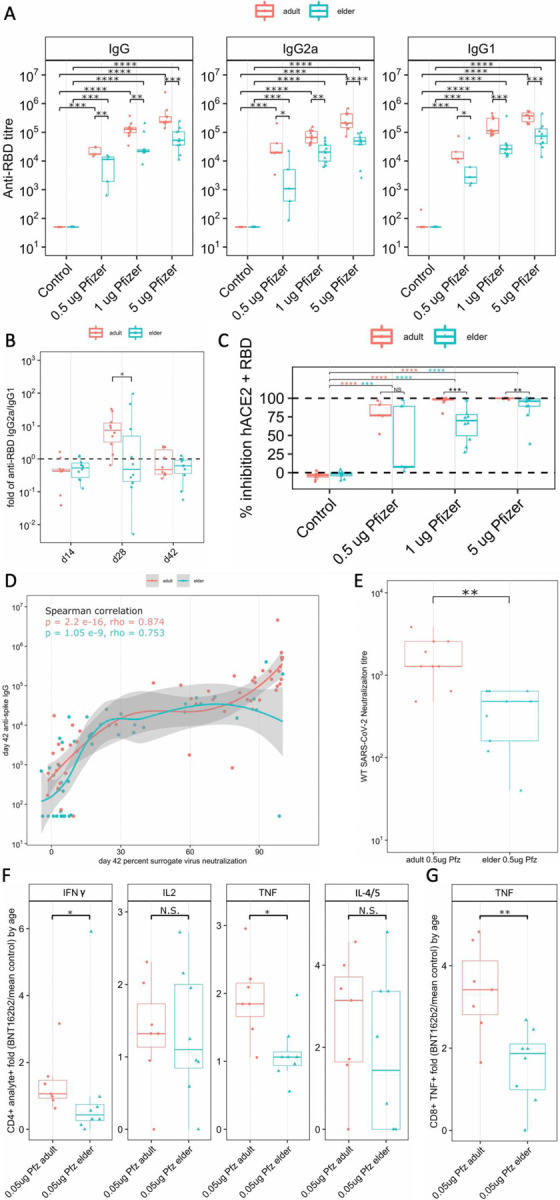
Reduced humoral and T_H_1 cellular immunogenicity of BNT162b2 vaccine in aged mice. Adult (6–12 weeks old, red) and Elder (>10 months old, blue) mice were prime boost immunized by intramuscular injection with 0.05 to 5 μg of BNT162b2 mRNA (Pfizer, Pfz). Humoral immunity to wildtype receptor binding domain (RBD) of SARS-CoV-2 spike antigen was quantified on Day 42 post-prime immunization with significant induction of (**A**) total IgG (left), IgG2a (middle), and IgG1 (right) in adults (red) and elders (teal). Elder mice demonstrated significantly lower Ab titers for each Ig isotype. T_H_-polarization was evaluated individually with IgG2a, a T_H_1 marker and IgG1, a T_H_2 marker. (**B**) T_H_ balance was also evaluated by fold-change IgG2a/IgG1 for periodic shifts in T_H_-polarization on Days 14, 28, and 42 post-prime immunization. (**C**) Ab efficacy was measured by a surrogate virus neutralization test (SVNT) measuring the ability of Ab to reduce RBD binding to human ACE2. A dose-dependent, elder-impairment of Ab efficacy was observed. (**D**) Correlation analyses of anti-spike IgG by SVNT was significantly correlated and similar in adult and elder mice. (**E**) Compared to adult mice, sera from aged mice were significantly impaired at neutralizing WA-1 SARS-CoV-2 in an *in vitro* live virus neutralization assay quantifying the dilution required to lose 99% neutralization of cytopathic effects against Vero TMPRSS2 cells. Cellular immunity evaluated by spike-specific peptide restimulation of splenocytes identified SARS-CoV-2 specific CD4^+^ and CD8^+^ T cell responses by flow cytometry, normalizing age groups by dividing by mean of control mice per age group. (**F**) CD4^+^ T cells were baseline-normalized age-stratification and calculating the fold increase of IFNγ positive cells of BNT162b2-immunized over the average of vehicle control mice. Cell positivity for IFNγ^+^, IL-2^+^, TNF^+^, and IL-4/5^+^ (left to right) was evaluated, with significantly lower T_H_1 polarized IFNγ^+^ and TNF^+^ responses in elder mice. (**G**) CD8^+^ T cell TNF^+^ cell positivity was evaluated, and significant elder impairment was observed. Sample sizes were (A-D) n = 5–10, (E) n = 9, and (F-G) n = 7–8. Normality was determined with Shapiro-wilk then significance evaluated in A-D with Kruskal-Wallis followed by one-sided Wilcoxon rank-sum tests, in E with one-sided Wilcoxon rank-sum test, in D with locally estimated scatterplot smoothing (loess) modelling, in F with two-sided Wilcoxon rank-sum test, and in G with two-sided T-test. Significance is denoted by * p < 0.05, ** p < 0.01, *** p < 0.001, **** p < 0.0001.

*In vivo* murine intramuscular BNT162b2 vaccination (Extended data Fig. 2A) validated age-specific observations. As observed in humans^25,26^, elder mice sera displayed significantly lower total immunoglobulin G (IgG), IgG2a, and IgG1 Ab immunogenicity, with lower anti-receptor binding domain (RBD) Ab titers than adult mice (Fig. 5A). Adult and elder mice displayed waning immunity between Days (D) 42 and 210 post-prime immunization, at various immunization doses (Extended data Fig. 2B–G). Mirroring human elder observations^36^, greater waning of immunity was observed in 1 μg-immunized elder mice, with 63–75% more waning immunity across IgG, IgG2a, and IgG1 based on the median fold change of D210 over D42 between age groups (Extended data Fig. 2H). A trend of 30–83% increased waning was observed at other immunization doses. Ab isotypes IgG2a and IgG1, respective markers of T_H_1 and T_H_2 polarized immunity^57^, were induced over nonvaccinated controls (Fig. 5A). The IgG2a/IgG1 ratio inferring T_H_1 (>1) or T_H_2 (<1) polarization identified a transient impairment of T_H_1 on D28 post-prime in elder mice (Fig. 5B). Ab function was inferred via sera inhibition of RBD binding to recombinant human angiotensin-converting enzyme 2 (hACE2) in a surrogate virus neutralization assay (sVNT), as a correlate of protection^58,59^. Elder mice had lower sVNT than adult mice at multiple immunization doses (Fig. 5C). A non-linear positive correlation of D42 total IgG Ab with sVNT was observed in both ages, with similar model fitting (rho) (Fig. 5D). Murine Ab neutralization of live Washington-1 (WA-1) SARS-CoV-2 *in vitro* demonstrated an impaired elder response compared to adult mice (Fig. 5E). Spike peptide splenocyte stimulation induced CD4^+^ T cell IFNγ, IL-2, TNF, and dual stained IL-4 and −5 positivity, alongside CD8^+^ TNF (Extended data Fig. 3, Table S3). Baseline population differences in CD4^+^ T cell populations were accounted for by dividing mouse BNT162b2-immunized responses by the average of age-matched vehicle control immunized mice. Elder mice had significantly less fold-induction of CD4^+^ T cell IFNγ and TNF cell positivity compared to adult mice (59% and 43% lower median fold induction, respectively, Fig. 5F). IL-2 was unchanged, while IL-4/5 was non-significantly trending lower in elder mice (54% lower median elder FC induction). Similarly, CD8^+^ TNF^+^ T cell fold induction was significantly impaired in elder vs. adult mice (45% less median elder FC, Fig. 5G). *In vivo* murine evaluation mirrored human results with age-associated impaired Ab production, Ab function, class switching, and CD4^+^ and CD8^+^ CMI.

## Discussion:

Herein, we have demonstrated for the first time that (a) human *in vitro* modeling of proteomic responses to mRNA vaccines is feasible, (b) such modeling demonstrates marked age-dependent differences; and (c) results *in vitro* can be verified in aged mice *in vivo*. While mRNA vaccines have been crucial in combatting the SARS-CoV-2 pandemic, much remains to be learned regarding mRNA vaccine-induced immune activation, and mechanisms contributing to age-associated decreased immunogenicity^26,30–32^. We hypothesized that elder participants would have differential immune activation post-BNT162b2 WBA stimulation skewing immune responses towards distinct functional states. As elders have higher rates of severe COVID-19^60^ and reduced vaccine immunogenicity^12,25,26,29,33,61^, understanding the mechanisms and contributing factors to weaker immunogenicity is an urgent unmet need that may inform future vaccine optimization, discovery and development. To our knowledge, we report the first combination of human *in vitro* WBA and murine *in vivo* systems modelling age-specific immune activation with the mRNA vaccine, BNT162b2.

Immune activation following BNT162b2 stimulation could be through the pattern recognition receptors toll like receptor (TLR)-2, TLR-3, TLR-4, TLR-7, TLR-8, retinoic acid-inducible gene I (RIG-I), and melanoma differentiation-associated protein 5 (MDA-5) recognizing multiple vaccine components^62–65^. Among other PRRs, SARS-CoV-2 can also activate MDA-5 signaling^66^, suggesting some similarity of sensory systems detecting mRNA vaccines and SARS-CoV-2 virions. Development of vaccine formulations that trigger similar innate immune activation as natural infection may enhance immunogenicity against microbial pathogens^10,67^. To assess similarities of BNT162b2 induced immunogenicity and SARS-CoV-2 infection, we evaluated BNT162b2-induced DEPs to proteins induced during infection, as described in literature. We employed two complementary proteomic approaches for the *in vitro* evaluation of BNT162b2-induced WBA responses. LC/MS proteomics identified DEPs of BNT162b2-stimulated WBA adult samples compared to vehicle control (Fig. 1). Adult samples, but not those from the elderly, had an induction of complement component C1S, the ATPase PSMC6, hemoglobin HBE1, and the metallo-protease CPN1 which have each been implicated in host response to SARS-CoV-2, COVID-19 severity, and/or have anti-viral activity^68–73^. Additionally, stimulated adult, but not elder, samples had reduced PPIA. Lower PPIA has been associated with better COVID-19 prognosis^74^, potentially due to the protein folding function of PPIA that may bind to SARS-CoV-2 mRNA^75^. Elder participants were markedly distinct from adults. There was only a single overlapping downregulated protein between the age groups, DEFA3 (Fig. 1A–D, 2A) which has been associated with lipid envelopes^76^. SAA1, FGG, and FGB were induced in elder BNT162b2-stimulated samples, but not adults, and have been associated with SARS-CoV-2 infection and/or worsened disease severity^77–80^. Several DEPs that are downregulated in elders, including TPM4, APOF, APOC2, CPN1, and PI16, were downregulated by exposure to SARS-CoV-2 virions in humans *in vitro* and *in vivo*, associating with poor prognosis^73,81–83^. Overlap of impaired elder BNT162b2 responses with factors associated with disease susceptibility may reflect important common signaling pathways shaped by immunosenescence. A secondary guided PEA-based proteomic assay validated results (Fig. 1E, F), observing a similar age-dependent pattern of BNT162b2-induced adult up and elder down regulation as the LC/MS proteomics, but with distinct analytes. The unsupervised heatmap clustering of 4/5 adult study participants (Fig. 1G) support consistent adult immune activation patterns, while lack of elder clustering further demonstrating broad age-related differences (Fig. 1H). The marked differences in early proteomic responses between adult and elder participants may contribute to differences in BNT162b2 immunogenicity.

Analysis of the proteome in supernatants derived from BNT162b2-stimulated adult vs. elder WBAs via LC/MS (Fig. 2B–D) and PEA (Fig. 2E–F) analyses demonstrated marked age-dependent differences. Multiplex analysis revealed that elders had impaired BNT162b2-stimulated chemokine CCL4 production (Fig. 2G), though functional categorization of multiplex-quantified chemokines did not identify broad differences in chemokine induction (Extended data Fig. 1). Significance of the single CCL4 chemokine, but not the chemokine functionally grouped analysis, could indicate specific immunoregulation but requires further validation and evaluation. CCL4 induction has been negatively correlated with age^13^, potentially impacting monocyte and antigen presenting cell (APC) chemotaxis to injection site and lymph nodes, respectively^84–87^. Network analysis of DEPs (Fig. 2H–I) further supported the overall lower elder activity. Contrasting adults, elders lacked the ‘*IL-4 and IL-13 signaling*’ network after BNT162b2 stimulation. IL-4 and IL-13 can polarize T cell differentiation towards T_H_2, support B cell differentiation, and induce class switching^88^, therefore node absence in elders could be another characteristic of mRNA vaccine immunosenescence. Elder participant samples had an additional pathway not observed in adults, “*immunoregulatory interactions between a lymphoid and non-lymphoid cell*” with predominantly impaired induction of analytes. One of the inferred genes, Cytotoxic and regulatory T cell molecule (*CRTAM*), can support CD4^+^ and CD8^+^ T cell differentiation^89^, such that downregulation in elders may reduce CMI.

A targeted multiplex assay quantifying cytokines and chemokines enabled analyses of functionally grouped analytes, yielding further insight. In the *in vitro* WBA, BNT162b2 induced concentration-dependent production of IL-6, CXCL8, TNF-α, and IFNγ (Fig. 3A), each of which have been associated with SARS-CoV-2 infection^90^, with IFNγ induction post-infection negatively correlated with advancing age^91^. Of the cytokines measured, IL-1β, IL-1RA, IL-6, TNF, CCL2, CCL3, and CCL4 (Fig. 4, Extended Data Fig. 1) have been similarly induced by *in vitro* adult human PBMC stimulations with a LNP encapsulating mRNA encoding non-SARS-CoV-2 antigens, sharing properties of the mRNA-1273 SARS-CoV-2 vaccine, particularly mRNA and the SM-102 cationic lipid (a component of Moderna’s mRNA-1273, but not BNT162b2)^92^. Commonalities of immune activation across mRNA vaccine platforms may reflect similarities in innate immune responses reflecting inherent self-adjuvantation.

BNT162b2-induced WBA cytokine and chemokine induction was age-dependent with impaired production of IL-1RA and the T_H_1 polarizing^93–99^ CXCL10, and IFNγ in elders (Fig. 3B). Expression of these analytes was consistent across each of the 3 LC/MS proteomics, PEA proteomics, and multiplex platforms increasing confidence of true positives. Impaired IFNγ production may be critical, as blocking IFNγ has impaired BNT162b2 responses in adult mice^100^. Adult humans have T_H_1-polarized immunity after BNT162b2 and mRNA-1273 immunization^101–103^. This adult T_H_1 polarization has been partly attributed to cationic lipids, like DOTAP (1,2-dioleyl-3-trimethylammonium-propane chloride salt) used in cancer RNA-LNP therapies^101^. This lipid differs from BNT162b2’s ALC-0315 ((4-hydroxybutyl)azanediyl)bis(hexane-6,1-diyl)bis(2-hexyldecanoate))^8^, and BNT162b2 benefits from the reduced cytotoxicity from ionizable lipids^8^. This could impact self-adjuvanticity between formulations, and requires further investigation, as the head groups of cationic lipids can be toxic^104^.

Impaired T_H_1 polarization has been observed following BNT162b2 immunization of the elderly^29^, and could reflect impaired production of polarizing cytokines, impaired naïve T cell frequency, reduced sensitivity to cytokines during differentiation, and/or impaired chemotaxis of APCs. We hypothesized functionally grouping analytes could identify age-dependent impairment or dysregulation of T_H_1, T_H_2, T_H_17, or T_reg_ polarizing, chemokine, hematopoiesis-supporting, or trained immunity associated analytes. Interpreting individual cytokines from a multiplex is difficult for polyfunctional analytes and can cause overinterpretation of results. Pairing individual analysis with functionally grouped analytes can generalize changes to describe broader differences and assist with analyte redundancies^105^. The 41 multiplex-quantified analytes were categorized by function following targeted literature searches (Table S2). Additional analytes were not categorized in Table S2, as they were not measured by the bead-based multiplex. Functional assignment required evidence of being a polarizing molecule, not from being produced by a polarized cell. GEEGLM analysis identified a significant reduction (average 7.4% across multiple analytes, p = 0.027) of T_H_1 polarizing analyte induction in elder WBA responses (Fig. 4). To reduce the risk of overinterpretation, we employed a conservative GEEGLM analysis that averaged the functionally grouped analytes, including those that were not individually induced, thereby biasing towards no difference. Significantly impaired T_H_1 polarizing capacity persisted in elder samples. A lack of significance in the other 6 functions could indicate no impairment for these endpoints, or sample size limitations. Analyte source was not evaluated in this study, but various cells could contribute to immunosenescence. Ontogeny affects dendritic cells, monocytes, natural killer, and T cells^106–108^, and additional investigation is needed to identify which specific cell types contribute to elder impairment in the context of mRNA vaccines. We present a decreased production of multiple analytes, particularly a decrease in those polarizing towards T_H_1, in human elder samples compared to adult samples.

Impaired cytokine responses in elders may result in dysregulated innate immune responses^38^, DC TLR function^109,110^, APC phagocytosis and chemotaxis^110^, T cell maturation, hematopoiesis, follicle activity, B cell maturation, alternative recruitment of pro-inflammatory cells during infection^111^ and/or higher T_reg_ frequencies^16,112^. Compared to healthy middle aged adults, BNT162b2 immunization of human elders demonstrates impaired CD4^+^ T cell response and increased expression of the Programmed cell death protein 1 (PD-1) immaturity marker in peripheral blood^113^. Impaired elder Ab isotypes IgG1 and IgG3 have been observed post-vaccination^114^, each markers of human T_H_1 polarization^43,44^. We present impaired human induction of T_H_1 polarizing IFNγ and CXCL10 (Fig. 3B), and a functional analysis identifying broad impairment of T_H_1-polarizing analyte responses (Fig. 4) that may contribute to the observed impaired elder T_H_1 responses. Other features of immunosenescence, such as reduced naïve T cell availability^115^ and thymus involution^116^, may still impact the impaired immunity observed in the elderly, and requires further investigation.

Investigating BNT162b2-induced immune activation *in vitro* offers significant insights into species (i.e., human)- and age-specific responses, but may not completely reflect relevant vaccine responses *in vivo*. To investigate age-dependent BNT162b2-induced responses *in vivo*, we employed an age-specific murine model widely used to study mRNA vaccination^117–119^. Coupling human *in vitro* modeling with animal models represents a powerful approach to provide both species-specific and *in vivo* assessments. Increased age is associated with impaired human humoral immunity following BNT162b2 or mRNA-1273 vaccination^2,26,36,120^. Aged mice demonstrated impaired Ab induction at all immunization doses (Fig. 5A), and waning immunity was more rapid in aged mice (Extended data Fig. 2), mirroring human observations^36,121^. Indirect evaluation of T_H_1/T_H_2 polarization was observed via obtaining the ratio of IgG2a and IgG1 Ab isotypes, markers of T_H_1 and T_H_2 polarization, respectively^57^. The relative ratio following first immunization of IgG2a/IgG1 was moderately T_H_2-shifted and not different between both age groups, while the post-booster was T_H_1-shifted in adult, but not aged, mice (Fig. 5B). The timing of elder mouse impaired T_H_1 polarization post-booster but not post-prime suggests potential booster specific impairments that could impact future booster campaigns. Ab efficacy was evaluated with sera neutralization capacity, an important correlate of protection^58,59,122,123^. Elder mice had impaired neutralization in both sVNT (Fig. 5C) and live-virus WA-1 SARS-CoV-2 assays (Fig. 5E), highlighting the vulnerability of this population in a controlled environment, and mirroring age-dependent human observations of neutralization^124^. The murine model results of lower elder anti-RBD Ab titer, reduced markers of T_H_1 polarization, and impaired neutralization capacity had each matched age-specific human observations. These results validate the age specificity of the murine model. sVNT significantly correlated with anti-spike IgG immunity in both age groups, suggesting that Ab quantity was the important neutralization factor (Fig. 5D). CMI can support humoral immunity development and induce cytotoxic immunity against infected cells. Impaired T_H_1-polarizing analytes and Ab isotypes enable inference of polarization, but directly observing T cells can provide additional insight. Stimulation of splenocytes with spike-specific peptide induced IFNγ and TNF in CD4^+^ T cells, markers of T_H_1 polarization^125^, with 43–59% less median induction of cell positivity in elder mice compared to adult mice (Fig. 5F). Additionally, elder mice had significant impairments in BNT162b2-induced TNF^+^ CD8^+^ T cells (Fig. 5G), an important cell subset for lysing infected cells^125^. Elder groups remain vulnerable due to insufficient immune activation culminating in impaired humoral and cellular immunogenicity. The employed murine model captured many age-specific responses observed in humans, including impaired T_H_1 polarization in elders. Reduced BNT162b2 protection of elders likely reflects multiple contributing factors including lower vaccine-induced Ab titers, impaired Ab neutralization capacity, diminished CD8^+^ T cell activity, and reduced T_H_1 polarization of CD4^+^ T cells, coupled with rapidly waning immunity.

Impaired elder immune activation from BNT162b2 identifies an inherent difference within this population. Multiple approaches may amplify elder immunogenicity, including: (a) dose-escalation^26^, which may be well tolerated given lower reactogenicity in elders^126^, (b) additional boosters to extend protection^122,127^, albeit temporarily, due to rapidly waning immunity in elders^36^, and (c) use of T_H_1-polarizing adjuvants targeted towards elder populations^22^, that can enhance effective intracellular pathogen responses^41,42^, B cell class switching (human IgG1 and IgG3^43,44^, or murine IgG2a^43^), and support T_FH_-independent B cell responses^45^, as successfully pursued with Alum:CpG, saponin or MF59 activation of elder immunity^10,128–131^.

Our study features multiple strengths, including (a) use of a human WBA *in vitro* that is replete with age-specific cellular and soluble factors that could reflect or predict vaccine responses *in vivo*^39,132^, (b) use of three complementary proteomic approaches (mass spectrometry, PEA and multiplex assay) to gain a comprehensive view of the impact of BNT162b2 on the WBA proteome, and (c) validation of findings using aged vs adult mice *in vivo*. The use of human *in vitro* assays is notable as such approaches enable species- and age-specific modeling with individuals serving as both control and test conditions permitting paired analyses of new and established/licensed vaccine formulations, thereby accelerating and de-risking vaccine discovery and development^39,40^.

As with any research effort, our study has multiple limitations, including (a) grouping individuals into adult (18–50Y) and elder (≥60Y), which is common^110,133–136^, but does not capture the gradient of immune impairments seen in >80Y^26,135^, or >100Y^137^, (b) age-dependent differences in vaccine uptake resulted in differential vaccination coverage between groups that could not be controlled for, (c) the *in vitro* WBA assay lacks fluid flow and interaction with other tissues (e.g., muscle) and may not completely reflect immune responses *in vivo*, (d) the WBA did not define the cellular origin of the T_H_1 polarizing cytokine and chemokines measured, and (e) immune proteins have redundancies^105^ that are progressively lost during immunosenescence^138^. We reduced the risk of over-interpreting the biologic significance of single analytes by evaluating impaired elder induction of both individual T_H_1 polarizing analytes, IFNγ and CXCL10^93–98^ (Fig. 3B), and the analysis of functionally grouped responses (Fig. 4). We further validated the observed *in vitro* responses in an *in vivo* aged mouse model (Fig. 5).

**Extended Data Fig. 4.**
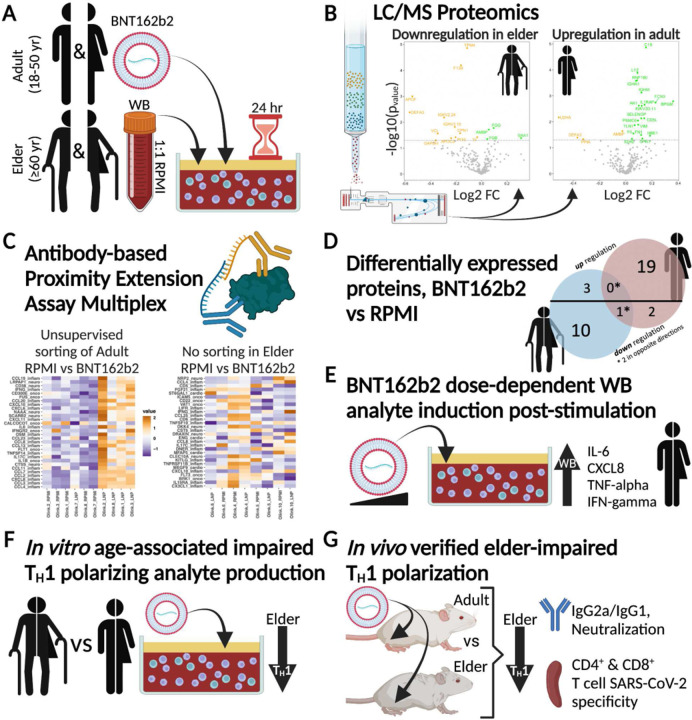
mRNA vaccines induce distinct age-dependent immune responses *in vitro* and *in vivo*. (**A**) Schematic of the stimulation of adult (18–50Y) and elder (≥60Y) whole blood assay (WBA) with the lipid nanoparticle encapsulated mRNA vaccine, BNT162b2. (**B**) LC/MS proteomics of WBA supernatant identified general downregulation in elders (left) and upregulation in adult (right). (**C**) Further WBA evaluation with Proximity Extension Assay (PEA) quantification of 368 proteins resulted in clustering of adult BNT162b2-stimulated samples apart from vehicle control RPMI (left), while elder participant samples (right) did not cluster on treatment. (**D**) Proteomics-identified differentially expressed proteins had distinct regulation patterns with adult-associated up regulation (above the horizontal black line) or elder-associated down regulation (below black line). (**E**) Quantification of cytokines and chemokines multiplexing identified dose-dependent induction of pro-inflammatory markers in adult samples, with age-associated impairments. (**F**) Grouping of *in vitro* produced analytes by functional category identified an age-associated decrease in T_H_1 polarization. (**G**) *In vivo* murine experiments with adult (6–12 weeks) and elder (>10 months) mice validated age-associated decreased humoral immunity, impaired markers of T_H_1 polarization (ratio of antibody subtypes IgG2a/IgG1), and decreased cell frequency of CD4^+^ and CD8^+^ T cells.

In summary, supernatants from adult and elder WBA (Extended data Fig. 4A) demonstrated distinct BNT162b2-induced immune activation patterns by LC/MS and PEA proteomics (Extended data Fig. 4B–C), with BNT162b2-induced adult upregulation and elder down regulation. DEP profiles were markedly age-dependent, with only 1 overlapping significant protein downregulated in both adults and elders (DEFA3, Extended data Fig. 4D). Cytokine and chemokine multiplex demonstrated a vaccine concentration-dependent response in human adults *in vitro*, including IL-6, CXCL8, TNFα and IFNγ production (Extended data Fig. 4E). Categorization and evaluation of mono- and polyfunctional analytes highlighted impaired T_H_1 polarizing analyte induction in elders (Extended data Fig. 4F), a potential contributing mechanism to reduced immunogenicity in this vulnerable population. Murine *in vivo* experiments mirrored findings *in vitro*, with aged mice demonstrating reduced IgG2a/IgG1 ratio and reduced CD4^+^ T cell T_H_1 polarization (Extended data Fig. 4G). Our study has demonstrated the value of a human *in vitro* platform to model age-specific responses to the mRNA vaccine BNT162b2. mRNA vaccines are expected to continue to be essential for combatting the on-going coronavirus pandemic and are additionally being evaluated for a range of infectious diseases (influenza, RSV, and HIV) and oncology^5,139^. Identifying critically divergent functions between age groups is an essential step for optimizing the next generation of precision mRNA vaccines to overcome immunosenescence. Accordingly, translational research is needed to enhance elder immune responses including expanded adjuvantation efforts to enhance T_H_1 polarization, durable immunogenicity, and associated protection^22^.

## Extended Data

## Supplementary Material

Supplement 1
