# Supplementary material for: The mRNA vaccine BNT162b2 demonstrates impaired TH1 immunogenicity in human elders in vitro and aged mice in vivo
Source: Res Sq. 2022 Dec 21:rs.3.rs-2395118. Preprint. [Version 1] doi: 10.21203/rs.3.rs-2395118/v1 (PMC9810224; doi:10.21203/rs.3.rs-2395118/v1)
Supplement: Supplement 1 [file NIHPPRS2395118v1-supplement-1.pdf]

Online Methods:

Human participant consent and sample processing

Samples were collected from younger and middle-aged adult (18-50Y), and elderly, older adults (≥60Y) study participants following informed consent, under a protocol reviewed and approved by Boston Children's Hospital (BCH) Institutional Review Board (protocol # X07-05-0223). Per Table S1, study participants were 41.7% (5/12) and 35.7% (5/14) female in adult and aged groups, respectively. Self-reported SARS-CoV-2 infection history, at time of sampling, was <10%. Age groups differed in the percent prime and boost-immunized at time of sampling: 33% of adults had 1-2 vaccinations and 78.6% of elders were twice vaccinated. Peripheral blood was obtained from healthy adults (18-50 years old) and elders (>60 years old). Participants were excluded if they had symptoms of an infection within the last 7 days, had anti-inflammatory medication, were less than 110 pounds, had donated blood or been immunized within the past week, were known to be pregnant or were known to be HIV infected. Blood was drawn with pyrogen-free heparin anti-coagulation used at 20 units/mL (American Pharmaceutical Partners Inc.). Whole blood assay (WBA) stimulation was performed as described [1,2] with a few modifications. Briefly, fresh blood was mixed 1:1 with RPMI 1640 (Gibco 11875-119, with L-glutamine) and pipette-mixed with stimuli in 96-well U-bottom tissue culture plates (Becton Dickinson) with a 250 µL final culture volume for 24-hour incubation at 5% CO_2 in a humidified 37 ^{o}C incubator. The WBA included stimulations with per-well totals of 0.003, 0.03, 0.3, 1.0, and 3.0 µg of mRNA encoding spike antigen contained within BNT162b2 that is 0.1 µg mRNA/µL, corresponding to final stimulation % vol/vol of 0.012, 0.12, 1.2, 4, and 12%, respectively, for 24 hours. Supernatant was collected by centrifuging (500g, 10 minutes), collecting the cell-free supernatant fraction. Aliquots were stored at -80 ^{o}C.

mRNA vaccine

In vitro (human) and *in vivo* (murine) studies employed the monovalent Pfizer/BioNTech BNT162b2 mRNA vaccine obtained from the BCH Pharmacy (collected between February 2020 through August 2022). All vaccine material used was left over from immunization clinic, i.e., residual overfill droplets remaining after removal of injectant for human immunization and was strictly material that would have otherwise been discarded. Material was used within 12 hours of vial puncture. Only monovalent BNT162b2 with mRNA encoding wild-type SARS-CoV-2 spike protein was used in this study.

Evaluation of human culture supernatant

In vitro response to Pfizer/BioNTech's BNT162b2 stimulation of the WBA was quantified by targeted plasma proteomics (liquid chromatography, mass spectrometry, LC/MS), by a proximity extension assay (PEA, 4x Olink Target 96™ platforms) proteomics, and by a multiplex bead-based assay investigating inflammation and chemotaxis mediators. Each assay is described below.

Targeted plasma proteomics sample preparation

WBA samples were evaluated by LC/MS proteomics observing a dose-titration of BNT162b2-stimulated whole blood (WB). All chemicals and reagents were purchased at the highest purities available. Solvents used in this study were LC/MS grade and purchased from Fisher Chemicals (Thermo Fisher Scientific). Briefly, a volume of 10 µL of 10-fold diluted plasma was mixed with 60 µL of urea buffer (8M urea in 50 mM ammonium bicarbonate, Sigma Aldrich) and 15 µL of dithiothreitol buffer (DTT, 50 mM in urea buffer, Sigma Aldrich) before being incubated for 30 min on a thermomixer (800 rpm, room temperature, RT). The samples were alkylated with iodoacetamide buffer (375 mM in urea buffer, Sigma Aldrich) and incubated for 30 min (800 rpm, RT and dark). A volume of 10 µL of DTT buffer was added to quench the alkylation. The samples were transferred to the SP3 beads mixture (Sera-Mag SpeedBeads, 1:1 v/v, GE Healthcare) previously washed with high performance liquid chromatography (HPLC)-grade water (Sigma Aldrich) at a 1:10 protein to bead ratio. A volume of 150 µL of absolute ethanol (Superlco) was added and incubated 15 min on a thermomixer (1,000 rpm at RT). The samples were placed on the magnetic rack and then the clear supernatant was removed. The beads were washed three cycles in 200 µL 80% ethanol. After the final washing step, the samples were trypsinized with 100 µL of trypsin buffer (Promega, 20 µg/ml in 50 mM ammonium bicarbonate) and placed on thermomixer (1,000 rpm, 2 hours, 37 ^{o}C). After digestion, samples were centrifuged to pulldown the liquid and placed on magnetic rack to collect the supernatant and were acidified with 2% v/v formic acid in HPLC water. The C18 cleanup was performed using a 96-well MACROSPIN C18 plate (TARGA, The NestGroup Inc.) and the tryptic peptides were eluted off the C18 particles using 40% ACN/0.1% FA. The samples were then dried and stored at -20 ^{o}C until LC/MS analysis. The samples were analyzed using a LC system (Nexera Mikros, Shimadzu) equipped with Capillary C18 column (0.2 x 100mm, 2.7 µm particle diameter, Shimadzu) coupled online to an 8060 triple quadrupole mass spectrometer instrument (Shimadzu). From each sample, 1 µg peptide quantity was separated using a non-linear gradient over 15-minute run time operated at 10 µL/min (5% solvent B for 0.2 min; 5 to 40% B for 10.3 min; 85% B for 1.5 min and 5% for 3 min). The final scheduling method was performed using the following parameters: 1.2 sec of maximum loop time with minimum dwell time of 2 msec and pause time of 1 msec, Q1 and Q3 resolution set at the 'unit' level.

Proximity Extension Assay (PEA)

Human *in vitro* WBA samples were also evaluated by a Proximity Extension Assay (PEA) with Olink^{TM} technology as in [3]. Proteins were labelled with a mixture of antibodies containing pairs of antibodies tagged with a DNA barcode that were able to recognize the same protein in the same assay well. Antibodies binding to the same target, in close proximity, could have their DNA tags hybridize, undergo DNA polymerase-dependent extension, subsequent PCR amplification, and next generation sequencing (NGS). The dual antibody binding yields greater specificity, and PCR amplification resulted in a highly sensitive assay to evaluate normalized protein expression (NPX). Four Target 96^{TM} panels (Inflammation, Cardiometabolic, Oncology III, and Neurology) were assayed by Olink under a service agreement on the stimulated supernatant samples evaluating a total of 368 proteins, while blinded to age group. Analyses were performed at BCH. These panels were baseline-normalized to each other to reduce inter-assay variability and were evaluated for differential (up/down regulation) normalized protein expression in samples stimulated with 3 µg of encapsulated BNT162b2 mRNA against vehicle

(RPMI) controls. PEA heatmap analysis was unsupervised to evaluate if patterns of LNP-induced proteins could differentiate stimulated from non-stimulated in adults and elders. Euclidean-clustering was applied to evaluate BNT162b2-stimulated adult clustering and elder non-clustering. The top 100 differentially expressed proteins were converted to Entrez IDs with the AnnotationDbi package and then enrichment analysis was performed with ReactomePA. Plotting of network interactions involved cnetplot and ggraph.

Bead based multiplex

Human samples from WBA were also evaluated by a bead-based multiplex platform measuring 41 analytes (Milliplex HCYTOMAG-60K) following the manufacturer's recommendations and excluding samples with insufficient bead counts (requiring ≥ 30 beads / analyte).

Functional categorization of analytes

Selection of individual significantly induced proteins increases the risk of interpretation bias from analyte polyfunctionality and the potential of false positives. We augmented the classical approach of individual analyte interpretations from multiplex assays by additionally analyzing based on functional categorization to evaluate if age significantly interacted with each function. Immunosenescence could be driven by differential production of analytes polarizing towards $CD4^+$ T helper cell (T_H) 1, T_H2, T_H17, and T_{reg} differentiation, and those supporting chemotaxis, hematopoiesis, and/or associated with secondary effects of vaccine (e.g., trained immunity, nonspecific effects). T_H1 polarized immune responses can trigger effective intracellular pathogen responses[4], including $CD8^+$ T cell-mediated immunity[5], B cell class switching[6,7] and induction of T_{FH}-like activity for effective B cell responses in the absence of T_{FH}[8]. T_H2 responses can support Ab production but can bias towards IgE Ab class switching with potential age-dependent differences[6,9]. T_H17 has been associated with B cell differentiation and class switching to IgA[10-12], with increased mucosal immunity[13]. Chemokine responses are critical for mounting an effective immune response[14], through both initial recruitment of monocytes to the vaccination site, and subsequent chemotaxis of mature antigen presenting cells (APC) to the draining lymph node[15-18]. T_{reg} can restrain germinal center reactions[19,20]. Hematopoiesis-inducing compounds could be important immunoregulators, as impaired hematopoiesis has been associated with reduced vaccine responsiveness in the elderly[21,22]. mRNA vaccines may also induce trained immunity[23,24]. Impact of age on each function was evaluated through a targeted multiplex cytokine and chemokine assay measuring 41 predominantly polyfunctional analytes.

A literature review was performed to classify each of the 41-plex measured analytes into the functional categories of T_H1, T_H2, T_H17, and T_{reg} polarizing, and chemokine, hematopoiesis, or vaccine associated trained immunity inducing functions. Particular attention to differentiate polarizing activity from analytes that were produced by polarized cells was performed. This review included various gene ontology (GO) terms including 'T cell differentiation' (GO: 0030217, sub-divided to T_H1 or T_H2 or T_H17 polarizing), 'T-helper 17 cell lineage commitment' (GO: 0072540), 'regulatory T cell number' (GO: 0045066), 'Chemokine' (GO: 0032602), 'Chemotaxis' (GO 0006935), and 'Hematopoiesis' (GO: 0030097). The literature review was supplemented by targeted searches requiring the functional term, and "polarizing" or

"polarized", in the case of T_H-polarizing activity, in the google scholar database. Evidence from human sources was prioritized but supplemented with murine where human observations were not available. Categorization into the $CD4^+$ T cell polarizing capacities required evidence of being required for polarization, or inducing polarization itself, rather than being induced by a polarized cell. The other evaluated functions included direct and indirect chemokine activity, hematopoiesis support or induction, and mediating secondary effects of vaccines.

Animals

All BALB/c animals (BALB/cAnNHsd) were purchased from Envigo and housed at Boston Children's Hospital (BCH). Procedures were approved under the Institutional Animal Care and Use Committee (IACUC) with the supervision of the Department of Animal Resources at Children's Hospital (ARCH) under protocol number 00001573. Female adult mice were used between 6-22 weeks of age, and elder female retired breeder mice were utilized within 43-59 weeks of age, a similar age group as used in other studies of immunosenescence[25], communally housed with similarly aged animals. Adult mice were sex-matched to the availability of the elder mice, which were all female. Ear clipping identification allowed mouse tracking and a guided treatment randomization to balance treatments across cages, thereby reducing variability between treatment groups.

Murine intramuscular injection

Mice were injected with mRNA vaccine (BNT162b2) in 50 µL inoculum to the mouse's right hind limb via intramuscular (IM) administration in either conscious or isoflurane-anesthetized mice. A prime-boost schedule was followed, separated by 14 days.

Murine SARS-CoV-2 specific antibody evaluation

At 14, 28, 42, and 210 days post-prime immunization animals were anesthetized under 3% isoflurane and had 100-200 µL of blood collected by retroorbital bleed into non-heparinized glass capillary tubes (Drummond Cat. 1-000-1000). Prompt expelling of blood into microcentrifuge tubes was followed by allowing samples to clot. Blood was centrifuged within 2 hours (1500g, 7.5 minutes), transferred to new microcentrifuge tubes, recentrifuged, and serum was aliquoted for storage at -80 ^oC.

Anti-spike and anti-RBD titers were evaluated by ELISA utilizing a previously described protocol[19]. In brief, flat-bottomed high-binding 96-well Corning plates (NY, catalogue 9018) were coated with 25 ng per well of SARS-CoV-2 wildtype sequence of recombinant RBD (GenBank MN975262.1, amino acids R319-K529) or 50 ng per well of recombinant spike (GenBank MN90894, amino acids M1-Q1208) glycoprotein. These proteins were produced with constructs consisting of a TwinStrepTag, an HRV3C cleavage site, and an 8XHisTag C-terminal modification from Aaron G. Schmidt from the Ragon Institute, and Barney S. Graham from the NIH Vaccine Research Center, respectively. Overnight incubation at 4 ^oC was followed by 0.05% Tween 20 in PBS-wash of plates, with subsequent 1% bovine serum albumin (BSA) blocking for 1 hr at RT. Serum samples were initially diluted 1:100 then 4-fold serially diluted to a dilution factor of 1.05E8, followed by incubation in the pre-coated plate for 2 hr at RT. Following 3 washes a 1 hr RT incubation with horse radish peroxidase (HRP)-conjugated goat anti-mouse

IgG, IgG2a, or IgG1 (purchased from Southern Biotech respective cat. 1036-05, 1081-05, 1071-05) was performed. Following 5 x 0.05% Tween 20 in PBS washes, RT tetramethylbenzidine (TMB, BD OptEIA substrate solution from BD Biosciences) was added for 5 minutes, then stopped with sulfuric acid, 2N H_2SO_4. Optical density (OD) was determined at 450 nm in a SpectraMax iD3 microplate reader (Molecular Devices). Assignment of antibody titer was calculated from the final dilution where TMB was over 3x background. Anything that did not register 3x background was assigned half the initial serum dilution of 100.

Murine surrogate virus neutralization titre (sVNT) evaluation

Murine sera were evaluated using a previously described protocol[19]. Flat-bottomed high-binding 96-well Corning plates (NY, catalogue 9018) were incubated with 100 ng recombinant human angiotensin-converting enzyme 2 (hACE2, Sigma-Aldrich) in PBS, per well, overnight at 4 ^oC. Following 3 x washed with 0.05% Tween 20 in PBS plates were blocked for 1 hr RT with 1% BSA. Sera were initially diluted 1:160, then incubated with 3 ng of RBD conjugated with an Fc fragment of IgG (RBD-Fc) for 1 hr at RT. Sample mixtures were transferred to the hACE2 coated plates alongside the positive control (PC) of serum samples without pre-incubation, and the negative control (NC) of 1% BSA in PBS alone. After 3 x washes with 0.05% Tween 20 in PBS plates were incubated for 1 hr at RT with anti-human IgG Fc with HRP-conjugation (Southern Biotech). After an additional 5 x washes plates were TMB-developed, H_2SO_4 stopped, and read at 450nm, as in the Ab evaluation. Percent inhibition was evaluated by calculating (1 – (Sample OD - NC OD)/(PC OD - NC OD)) x 100.

Live SARS-CoV-2 virus neutralization test

Murine samples were evaluated as in [26,27]. Processing and scoring of samples were performed while blinded to treatment groups. Sera were heat-inactivated at 56 °C for 30 min to deactivate complement. Once equilibrated to RT, samples were processed in duplicate to evaluate neutralization titer. Samples were initially diluted 1:20, followed by a 1:2 serial dilution resulting in a 12-dilution series with each well containing 60 µL. Dilutions employed Dulbecco's Modified Eagle Medium (DMEM, Quality Biological) supplemented with 10% (v/v) heat-inactivated fetal bovine serum (HI-FBS, Gibco), 1% penicillin/streptomycin (v/v, Gemini Bio-products) and 1% L-glutamine (v/v, 2mM final concentration, Gibco). Dilution plates were transported to biosafety level (BSL)-3 where 60 µL of diluted SARS-CoV-2 inoculum (WA-1 strain, Courtesy of Dr. Natalie Thornburg, and the CDC) was added to each well with serum, resulting in a multiplicity of infection of 0.01, corresponding to 100 pfu/well. Each plate had a non-treated virus-only control and a mock-infection well to establish cytopathic effects. After 1 hr incubation at 37 ^oC with 5% CO_2, 100 µL of sample-virus complexes were transferred to a 96-well plate with confluent (~1e4) Vero TMPRSS2 cells. Incubation of cells with virus permitted evaluation of cytopathic effect (CPE) after 72 hr where the first dilution displaying CPE was set as the minimum sample dilution needed to neutralize >99% of the SARS-CoV-2 tested.

Murine splenocyte evaluation

For cell mediated immunity evaluation of murine responses from adult and older mice was evaluated by CO_2-euthanizing mice between days 39 and 41 post-prime immunization with prompt aseptic collection of spleens at 4 ^oC in 1 mL of RPMI 1640 (Gibco 11875-119) with 10%

HI-FBS (HyClone, GE Healthcare) that was 0.22 µm-filtered. Mouse euthanasia was batched to reduce the amount of time that the spleen was within the mouse without active circulation. Downstream splenocyte processing was batched with no more than 3 mice at a time to reduce the amount of time that cells were without circulatory support and off of ice. Aseptic dissection included care to dissect away pancreatic tissue, which otherwise can impact cell viability. Splenocytes were dissociated by gently pressing the spleen through a 70 µm cell strainer (Falcon cat. 352350) using the plastic portion of a 3 mL syringe's plunger, aseptically removed from its wrapper. After twice rinsing the strainer and plunger with 1 mL cold RPMI (4 ^{o}C), an additional 16 mL rinse of the strainer alone was performed. Following centrifugation (315g for 10 min) supernatant was decanted so that ≤ 200 µL of liquid remained, cells were resuspended in residual volume, and red blood cells (RBCs) lysed with 1 mL of RT Ammonium-Chloride-Potassium (ACK) lysis buffer (Gibco, Cat A10492-01, Waltham, MA) for exactly 2 minutes at RT. Osmotic lysis was neutralized immediately and cells washed with 25 mL cold RPMI, passed through a new 70 µm cell strainer, centrifuged, resuspended in RPMI + 10% HI-FBS, and cells were counted by dual Acridine Orange/Propidium Iodide (AOPI) staining (Nexcelom Cellometer K2, CS2-0106). Cells were plated at 2 x 10^6 total cells / well in 200 µL in a 96 well U-bottom plate, then rested overnight (37 ^{o}C, 5% CO_2) in T cell media to restore basal activity levels. T cell media consisted of RPMI 1640 (Gibco, Waltham, MA) supplemented with 10% HI-FBS (HyClone, Cytiva), 100 U/mL Penicillin and 100 mg/mL Streptomycin (Gibco, Waltham, MA), 55 mM 2-mercaptoethanol (Gibco, Waltham, MA), 60 mM non-essential Amino Acids (Gibco, Waltham, MA), 11 mM HEPES (Gibco, Waltham, MA), and 800 mM L-Glutamine (Gibco, Waltham, MA).

Flow cytometry of murine splenocytes

Following overnight rest, processed splenocytes were stimulated with SARS-CoV-2 wild type spike peptide pools (PepTivator, #130-126-700, Miltenyi Biotec) at 1 µg/mL in the presence of anti-mouse CD28/49d (1 µg/mL, BD) and brefeldin A (5 µg/ml, BioLegend). After 6hr of stimulation, cells were washed twice with PBS and blocked with Mouse Fc Block (BD Biosciences) according to the manufacturer's instructions. After blocking, cells were washed once with PBS and stained with Aqua Live/Dead stain (Life Technologies, Carlsbad, CA) for 15 min at RT. Following two additional PBS washes, cells were resuspended in 100 µL of FACS buffer (PBS supplemented with 0.2% BSA (Sigma-Aldrich)) containing mouse specific cell surface markers for flow cytometry. Markers included anti-mouse CD44 PerCP-Cy5.5, CD3 BV785, CD4 APC/Fire750 and CD8 BUV395. Details of the clone and manufacturer of each marker used in a customized nine color, 10 marker flow-cytometry panel are documented in Supplementary Table 3, and as in [28]. Cells were incubated with surface markers for 30 min at 4 ^{o}C. Cells were PBS-washed and fixed/permeabilized with a Cytofix/Cytoperm kit (BD, #554714), following manufacturer's recommendations. Cells were washed in 1X perm/wash solution and subjected to intracellular staining (30 min at 4 ^{o}C) using a cocktail of the following Ab: anti-mouse IFNγ Alexa Fluor 488, TNF PE Cy7, IL-2 PE, IL-4 BV421 and IL-5 BV421 in 1X perm/wash solution. Finally, cells were washed in PBS and fixed in PBS containing 1% paraformaldehyde (Electron Microscopy Sciences, Hatfield, PA) for 20 min at 4 ^{o}C. After two final washes in PBS, the cells were resuspended in PBS and stored at 4 ^{o}C until acquisition. Samples were acquired on a BD LSRFortessa (BD Biosciences; San Jose, CA) configured with blue (488 nm), yellow/green (568 nm), red (640 nm), violet (407 nm), and ultraviolet (355 nm) lasers using standardized good

clinical laboratory practice procedures to minimize variability of data generated. Analysis was performed using FlowJo software, v.10.8.1 according to the gating strategy outlined in Extended Data Fig. 3. Positive gates for each cytokine were determined using fluorescence minus one (FMO) controls for IFNγ, TNF, IL-2 and IL-4/5 where all antibodies were used except the targeted one. Population gating was performed blinded to treatment group. Baseline $CD4^+$ T cell activation can be impacted by inflammaging[29], therefore we performed baseline normalization by evaluating adult and elder mice for their fold induction of $CD4^+$ T cell responses from immunized mice over the average age-matched vehicle control.

Data processing, analysis, and graphing

Raw data from LC/MS proteomics was exported into Skyline software (v20.2.1.315)[30] for peak area and retention time refinement. Peptide intensity (average of transition pairs) and the protein abundance (average of peptide intensities) in all samples were exported from Skyline. The means of the peptide intensities were used for protein abundance, exported for analysis in R.

The dual-antibody dependent protein labelling and proximity extension assay (PEA) was evaluated by Olink with normalized protein expression (NPX) forwarded to BCH. BCH data formatting was performed with R (versions 3.3.2 and 4.1.1) and R studio (versions '1.3.1093' and '2022.02.3 plus 492'). This included handling of missing data for PEA assays where samples below lower limit of quantification (LLOQ) and samples that were NA (no data) were replaced with the limit of quantification values provided by, and as recommended by, Olink. Notably, in PEA assays 39 of 368 measured analytes had >60 % missing data across the three evaluated treatment groups, i.e., RPMI, R848 (positive control TLR7/8 agonist), and LNP. These were filtered out before downstream analyses. Due to the warning status in the quality control assessment by Olink, some samples were removed for one research participant in the elder age group.

Proteomic responses were analyzed with R with R studio to calculate fold change of stimulated samples divided by matched controls, for data reformatting, graphing, and data analysis. Specific statistical tests are indicated in figure legends. Titratable up- and down-regulated protein responses from LC/MS proteomics was evaluated by generalized estimating equations linear mixed model (GEEGLM) analysis[31-33] simplifying multiple doses into 1 measure and to determine if significant interacting relationships of stimulation and age impacted analyte level. Nominal p-values < 0.05, were observed. Significance of NGS responses from antibody-dependent PEA assays were evaluated by a moderated T-test between the WBA stimulated with the top concentration, 12 µg/mL, corresponding to 3 µg of mRNA encapsulated in BNT162b2, compared to matching vehicle (RPMI) control, reporting an adjusted P-value by Benjamini-Hochberg procedure. Multilevel principal component analysis on NPX was performed using the pca function in mixOmics 6.16.3 package. Correlation assays of NGS sequenced samples were combined into 1 measure of LNP-stimulated vs RPMI mock-stimulation, NPX on one axis against age as a continual variable with the top 3 cytokines based on adult vs elder (LNP) comparison reported.

Bead-based multiplex samples were evaluated by linear modelling testing for dose dependency of each analyte in non-transformed pg/mL. Calculation of fold change (FC) by dividing each stimulated sample by a matched RPMI control with $\log_{10}$ transformation of FC for normalization was performed. Subsequent GEEGLM was used to evaluate the interacting effect of age on various cytokine functions with non-interacting effect of stimulation by evaluating 'antigen dose' and 'age group' variable effects, without interaction, on LogFC. Generalized estimating equations linear mixed model (GEEGLM) required data completeness, and any samples with a missing analyte from one of the categorized functions, or from the vehicle control or BNT162b2 stimulation concentrations were excluded. The percent difference was interpreted from the summary of GEEGLM objects through natural base exponentiation of the point estimate, with the confidence interval determined by adding or subtracting 1.96 multiplied by the standard error, and data was presented by radar plot per-functional category, filtering for only the analytes associated with that function. Samples under the lower limit of detection were set to the lower limit of detection. Throughout the article figures with boxplots display the center line as median of each group, with the box covering the interquartile range (IQR). The whiskers describe the largest and smallest values no greater than 1.5 * the IQR. Some figures were created with BioRender.com to visualize kinetics and responses.

Data availability

Deidentified quality assured human data from this study <will be> deposited in repository ImmPort under accession # <pending>. Further inquiries could be directed to the corresponding author. Murine data will be made available upon requests submitted to the corresponding author.

Code availability

Data analysis in this manuscript was performed in R and R studio, as summarized above. Authors will provide specific codes for analyses and graphing to those who make a request of the corresponding author.

Supplementary Table 1: Meta clinical data for human study participants

	Adult	Elder
Number of participants	12	14
Age: median (range)	26 (20 - 48)	67 (63 - 71)
Sex (% female)	41.7%	35.7%
Percent with history of COVID infection (self-reported; % reported infection, % not reported, % not disclosed)	8%, 59%, 33%	7%, 93%, 0%
Percent immunized (self-reported, none, 1st, 2nd, not disclosed)	33%, 25%, 8%, 33%	21%, 0%, 79%, 0%

Supplementary Table 2: Analyte functional grouping, with 1 denoting associated role and 0 denoting lack of active polarization or functional support.

analyte	T_H1 polarizing	T_H2 polarizing	T_H17 polarizing	T_{reg} polarizing	Hematopoiesis	Chemokine	Trained Immunity	Supplementary Reference
CCL2	1	1	1	0	0	1	0	34-41
CCL3	1	0	0	0	0	1	0	35,41-46
CCL4	1	0	0	0	0	1	0	35,41,47-51
CCL5	1	1	1	0	1	1	0	35,41,52-61
CCL7	0	0	0	0	0	1	0	62-64
CCL22	0	0	1	0	0	1	0	52,65-71
CX3CL1	1	0	1	1	0	1	0	41,72-78
CXCL1	0	0	1	0	1	1	0	41,46,79-82
CXCL8	0	0	1	1	0	1	0	44,83-88
CXCL10	1	0	1	0	1	1	0	41,89-93
EGF	1	0	0	0	1	1	0	94-99
Eotaxin	0	0	0	0	0	1	0	100-105
FGF-2	0	0	0	0	1	0	0	106-108
Flt-3L	0	0	0	1	1	0	0	109-111
G-CSF	0	1	0	1	1	1	1	112-118
GM-CSF	0	0	1	1	1	1	1	119-131
IFN-α2	1	0	1	1	1	0	0	132-138
IFN-γ	1	0	0	1	1	1	0	139-148
IL-1α	1	1	1	0	1	1	0	149-155
IL-1β	0	0	1	0	1	1	1	127,128,151,156-164
IL-2	1	1	0	1	1	0	0	20,165-173
IL-3	0	1	0	0	1	0	1	119,174-178
IL-4	0	1	0	1	1	1	0	145,179-191
IL-5	0	0	0	0	1	1	0	105,167,181,192,193
IL-6	0	1	1	0	1	0	0	179,194-198
IL-7	1	1	1	1	1	0	0	199-206
IL-9	0	1	1	0	1	1	0	167,207-215
IL-10	0	0	0	1	1	0	0	140,216-220
IL-12p40	1	1	0	0	1	0	0	221-227
IL-12p70	1	0	0	0	1	0	0	140,228,229
IL-13	0	1	0	0	1	0	0	181,230
IL-15	1	0	0	0	1	0	0	231-236
IL-17A	0	1	1	0	1	1	0	13,237-241
IL-1RA	0	0	0	1	0	0	0	242-244
PDGF-AA	0	0	0	0	0	1	0	245-248
PDGF-AB/BB	0	0	0	0	1	1	0	246,249-253
sCD40L	1	0	1	1	1	0	0	254-266
TGF-α	0	0	1	0	0	0	0	267-270
TNF-α	1	1	1	0	0	0	0	271-288
TNF-β	0	0	1	1	0	0	0	46,288-294
VEGF	1	1	1	1	1	1	0	295-311

Acronyms: T helper (T_H), regulatory T cell (T_{reg}), C-C Motif Chemokine Ligand (CCL-), C-X-C Motif Chemokine Ligand (CXCL-), epidermal growth factor (EGF), Fibroblast Growth Factor (FGF), FMS-related tyrosine kinase 3 ligand (Flt-3L), Granulocyte Colony Stimulating Factor (G-CSF), Granulocyte-macrophage colony-stimulating factor (GM-CSF), interferon (IFN), interleukin (IL-), receptor antagonist (RA), Platelet-derived growth factor (PDGF), soluble Cluster of Differentiation 40 ligand (sCD40L), Transforming growth factor (TGF), Vascular endothelial growth factor (VEGF).

Supplementary Table 3: Panels and reagents for flow cytometry assay

Assay	Target	Clone	Fluorochrome	Vendor	Identifier	Dilution
	Viability	---	Brilliant Violet 510	Invitrogen	L34966	1:500
	CD3	17A2	Brilliant Violet 785	BioLegend	100232	1:40
T-Cell Cytokines (Mice)	CD4	RM4-5	APC/Fire 750	BioLegend	100568	1:160
	CD8	53-6.7	Brilliant UltraViolet 395 (BUV395)	BD Biosciences	563786	1:80
	CD44	IM7	PerCP-Cy5.5	BioLegend	103032	1:160
	IFNγ	XMG1.2	Alexa Fluor 488	BioLegend	505813	1:160
	IL-2	JES6-5H4	PE	BioLegend	503808	1:40
	TNF	MP6-XT22	PE Cy7	BioLegend	506324	1:160
	IL-4	11B11	BV421	BioLegend	504119	1:40
	IL-5	TRFK5	BV421	BioLegend	504311	1:160

References:

1 van Haren, S. D. *et al.* In vitro cytokine induction by TLR-activating vaccine adjuvants in human blood varies by age and adjuvant. *Cytokine* **83**, 99-109 (2016). https://doi.org:10.1016/j.cyto.2016.04.001

2 Kollmann, T. R. *et al.* Neonatal innate TLR-mediated responses are distinct from those of adults. *J Immunol* **183**, 7150-7160 (2009). https://doi.org:10.4049/jimmunol.0901481

3 Vaes, R. D. W. *et al.* Identification of Potential Prognostic and Predictive Immunological Biomarkers in Patients with Stage I and Stage III Non-Small Cell Lung Cancer (NSCLC): A Prospective Exploratory Study. *Cancers (Basel)* **13** (2021). https://doi.org:10.3390/cancers13246259

4 Raphael, I., Nalawade, S., Eagar, T. N. & Forsthuber, T. G. T cell subsets and their signature cytokines in autoimmune and inflammatory diseases. *Cytokine* **74**, 5-17 (2015). https://doi.org:10.1016/j.cyto.2014.09.011

5 Ekkens, M. J. *et al.* Th1 and Th2 cells help CD8 T-cell responses. *Infect Immun* **75**, 2291-2296 (2007). https://doi.org:10.1128/IAI.01328-06

6 Kawasaki, Y. *et al.* Evaluation of T helper-1/-2 balance on the basis of IgG subclasses and serum cytokines in children with glomerulonephritis. *Am J Kidney Dis* **44**, 42-49 (2004). https://doi.org:10.1053/j.ajkd.2004.03.029

7 Patil, H. P. *et al.* Antibody (IgA, IgG, and IgG Subtype) Responses to SARS-CoV-2 in Severe and Nonsevere COVID-19 Patients. *Viral Immunol* **34**, 201-209 (2021). https://doi.org:10.1089/vim.2020.0321

8 Miyauchi, K. *et al.* Protective neutralizing influenza antibody response in the absence of T follicular helper cells. *Nat Immunol* **17**, 1447-1458 (2016). https://doi.org:10.1038/ni.3563

9 Castle, S., Uyemura, K., Wong, W., Modlin, R. & Effros, R. Evidence of enhanced type 2 immune response and impaired upregulation of a type 1 response in frail elderly nursing home residents. *Mech Ageing Dev* **94**, 7-16 (1997). https://doi.org:10.1016/s0047-6374(96)01821-0

10 Milpied, P. J. & McHeyzer-Williams, M. G. High-affinity IgA needs TH17 cell functional plasticity. *Nat Immunol* **14**, 313-315 (2013). https://doi.org:10.1038/ni.2567

11 Christensen, D., Mortensen, R., Rosenkrands, I., Dietrich, J. & Andersen, P. Vaccine-induced Th17 cells are established as resident memory cells in the lung and promote local IgA responses. *Mucosal Immunol* **10**, 260-270 (2017). https://doi.org:10.1038/mi.2016.28

12 Shi, G. *et al.* Phenotype switching by inflammation-inducing polarized Th17 cells, but not by Th1 cells. *J Immunol* **181**, 7205-7213 (2008). https://doi.org:10.4049/jimmunol.181.10.7205

13 Kumar, P., Chen, K. & Kolls, J. K. Th17 cell based vaccines in mucosal immunity. *Curr Opin Immunol* **25**, 373-380 (2013). https://doi.org:10.1016/j.coi.2013.03.011

14 Mitchell, L. A., Henderson, A. J. & Dow, S. W. Suppression of vaccine immunity by inflammatory monocytes. *J Immunol* **189**, 5612-5621 (2012). https://doi.org:10.4049/jimmunol.1202151

15 Liu, J., Zhang, X., Cheng, Y. & Cao, X. Dendritic cell migration in inflammation and immunity. *Cell Mol Immunol* **18**, 2461-2471 (2021). https://doi.org:10.1038/s41423-021-00726-4

16 Lee, A. *et al.* A molecular atlas of innate immunity to adjuvanted and live attenuated vaccines, in mice. *Nat Commun* **13**, 549 (2022). https://doi.org:10.1038/s41467-022-28197-9

17 Simons, R. J. & Reynolds, H. Y. Altered immune status in the elderly. *Semin Respir Infect* **5**, 251-259 (1990).

18 Cunha, L. L., Perazzio, S. F., Azzi, J., Cravedi, P. & Riella, L. V. Remodeling of the Immune Response With Aging: Immunosenescence and Its Potential Impact on COVID-19 Immune Response. *Front Immunol* **11**, 1748 (2020). https://doi.org:10.3389/fimmu.2020.01748

19 Nanishi, E. *et al.* An aluminum hydroxide:CpG adjuvant enhances protection elicited by a SARS-CoV-2 receptor binding domain vaccine in aged mice. *Sci Transl Med* **14**, eabj5305 (2022). https://doi.org:10.1126/scitranslmed.abj5305

20 Rocamora-Reverte, L., Melzer, F. L., Wurzner, R. & Weinberger, B. The Complex Role of Regulatory T Cells in Immunity and Aging. *Front Immunol* **11**, 616949 (2020). https://doi.org:10.3389/fimmu.2020.616949

21 Agrawal, S. & Gupta, S. TLR1/2, TLR7, and TLR9 signals directly activate human peripheral blood naive and memory B cell subsets to produce cytokines, chemokines, and hematopoietic growth factors. *J Clin Immunol* **31**, 89-98 (2011). https://doi.org:10.1007/s10875-010-9456-8

22 Chen, C., Liu, Y., Liu, Y. & Zheng, P. mTOR regulation and therapeutic rejuvenation of aging hematopoietic stem cells. *Sci Signal* **2**, ra75 (2009). https://doi.org:10.1126/scisignal.2000559

23 Ziogas, A. & Netea, M. G. Trained immunity-related vaccines: innate immune memory and heterologous protection against infections. *Trends Mol Med* **28**, 497-512 (2022). https://doi.org:10.1016/j.molmed.2022.03.009

24 Giacomini, E. *et al.* Infection of human macrophages and dendritic cells with Mycobacterium tuberculosis induces a differential cytokine gene expression that modulates T cell response. *J Immunol* **166**, 7033-7041 (2001). https://doi.org:10.4049/jimmunol.166.12.7033

25 Roberts, A. *et al.* A mouse-adapted SARS-coronavirus causes disease and mortality in BALB/c mice. *PLoS Pathog* **3**, e5 (2007). https://doi.org:10.1371/journal.ppat.0030005

26 Nanishi, E. *et al.* mRNA booster vaccination protects aged mice against the SARS-CoV-2 Omicron variant. *Commun Biol* **5**, 790 (2022). https://doi.org:10.1038/s42003-022-03765-3

27 Gorman, M. J. *et al.* Collaboration between the Fab and Fc contribute to maximal protection against SARS-CoV-2 in nonhuman primates following NVX-CoV2373 subunit vaccine with Matrix-M vaccination. *preprint at bioRxiv* (2021). https://doi.org:10.1101/2021.02.05.429759

28 Barman, S. *et al.* Shaping Neonatal Immunization by Tuning the Delivery of Synergistic Adjuvants via Nanocarriers. *ACS Chem Biol* **17**, 2559-2571 (2022). https://doi.org:10.1021/acschembio.2c00497

29 Fransen, F. *et al.* Aged Gut Microbiota Contributes to Systemical Inflammaging after Transfer to Germ-Free Mice. *Front Immunol* **8**, 1385 (2017). https://doi.org:10.3389/fimmu.2017.01385
30 MacLean, B. *et al.* Skyline: an open source document editor for creating and analyzing targeted proteomics experiments. *Bioinformatics* **26**, 966-968 (2010). https://doi.org:10.1093/bioinformatics/btq054
31 Højsgaard, S., Halekoh, U. & Yan, J. The R Package geepack for Generalized Estimating Equations. *Journal of Statistical Software* **15**, 1 - 11 (2005). https://doi.org:10.18637/jss.v015.i02
32 Buzkova, P., Brown, E. R. & John-Stewart, G. C. Longitudinal data analysis for generalized linear models under participant-driven informative follow-up: an application in maternal health epidemiology. *Am J Epidemiol* **171**, 189-197 (2010). https://doi.org:10.1093/aje/kwp353
33 Prentice, R. L. & Zhao, L. P. Estimating equations for parameters in means and covariances of multivariate discrete and continuous responses. *Biometrics* **47**, 825-839 (1991).
34 Gu, L. *et al.* Control of TH2 polarization by the chemokine monocyte chemoattractant protein-1. *Nature* **404**, 407-411 (2000). https://doi.org:10.1038/35006097
35 Luther, S. A. & Cyster, J. G. Chemokines as regulators of T cell differentiation. *Nat Immunol* **2**, 102-107 (2001). https://doi.org:10.1038/84205
36 Kyriakides, T. R. *et al.* The CC chemokine ligand, CCL2/MCP1, participates in macrophage fusion and foreign body giant cell formation. *Am J Pathol* **165**, 2157-2166 (2004). https://doi.org:10.1016/S0002-9440(10)63265-8
37 Huang, D. R., Wang, J., Kivisakk, P., Rollins, B. J. & Ransohoff, R. M. Absence of monocyte chemoattractant protein 1 in mice leads to decreased local macrophage recruitment and antigen-specific T helper cell type 1 immune response in experimental autoimmune encephalomyelitis. *J Exp Med* **193**, 713-726 (2001). https://doi.org:10.1084/jem.193.6.713
38 Kara, E. E. *et al.* CCR2 defines in vivo development and homing of IL-23-driven GM-CSF-producing Th17 cells. *Nat Commun* **6**, 8644 (2015). https://doi.org:10.1038/ncomms9644
39 Morrison, B. E., Park, S. J., Mooney, J. M. & Mehrad, B. Chemokine-mediated recruitment of NK cells is a critical host defense mechanism in invasive aspergillosis. *J Clin Invest* **112**, 1862-1870 (2003). https://doi.org:10.1172/JCI18125
40 Bakos, E. *et al.* CCR2 Regulates the Immune Response by Modulating the Interconversion and Function of Effector and Regulatory T Cells. *J Immunol* **198**, 4659-4671 (2017). https://doi.org:10.4049/jimmunol.1601458
41 Laufer, J. M. & Legler, D. F. Beyond migration-Chemokines in lymphocyte priming, differentiation, and modulating effector functions. *J Leukoc Biol* **104**, 301-312 (2018). https://doi.org:10.1002/JLB.2MR1217-494R
42 Bhavsar, I., Miller, C. S. & Al-Sabbagh, M. Macrophage Inflammatory Protein-1 Alpha (MIP-1 alpha)/CCL3: As a Biomarker. *General Methods in Biomarker Research and their Applications*, 223-249 (2015). https://doi.org:10.1007/978-94-007-7696-8_27

43 Kang, J. G. *et al.* Zinc finger protein tristetraprolin interacts with CCL3 mRNA and regulates tissue inflammation. *J Immunol* **187**, 2696-2701 (2011). https://doi.org:10.4049/jimmunol.1101149

44 Broxmeyer, H. E. Regulation of hematopoiesis by chemokine family members. *Int J Hematol* **74**, 9-17 (2001). https://doi.org:10.1007/BF02982544

45 Staversky, R. J. *et al.* The Chemokine CCL3 Regulates Myeloid Differentiation and Hematopoietic Stem Cell Numbers. *Sci Rep* **8**, 14691 (2018). https://doi.org:10.1038/s41598-018-32978-y

46 Kimura, A., Naka, T. & Kishimoto, T. IL-6-dependent and -independent pathways in the development of interleukin 17-producing T helper cells. *Proc Natl Acad Sci U S A* **104**, 12099-12104 (2007). https://doi.org:10.1073/pnas.0705268104

47 Liu, J. Y. *et al.* CTL- vs Treg lymphocyte-attracting chemokines, CCL4 and CCL20, are strong reciprocal predictive markers for survival of patients with oesophageal squamous cell carcinoma. *Br J Cancer* **113**, 747-755 (2015). https://doi.org:10.1038/bjc.2015.290

48 Bystry, R. S., Aluvihare, V., Welch, K. A., Kallikourdis, M. & Betz, A. G. B cells and professional APCs recruit regulatory T cells via CCL4. *Nat Immunol* **2**, 1126-1132 (2001). https://doi.org:10.1038/ni735

49 Lim, H. W., Lee, J., Hillsamer, P. & Kim, C. H. Human Th17 cells share major trafficking receptors with both polarized effector T cells and FOXP3+ regulatory T cells. *J Immunol* **180**, 122-129 (2008). https://doi.org:10.4049/jimmunol.180.1.122

50 Burnett, A. *et al.* Angiopoietin-1 enhances neutrophil chemotaxis in vitro and migration in vivo through interaction with CD18 and release of CCL4. *Sci Rep* **7**, 2332 (2017). https://doi.org:10.1038/s41598-017-02216-y

51 Gauzzi, M. C. *et al.* Suppressive effect of 1alpha,25-dihydroxyvitamin D3 on type I IFN-mediated monocyte differentiation into dendritic cells: impairment of functional activities and chemotaxis. *J Immunol* **174**, 270-276 (2005). https://doi.org:10.4049/jimmunol.174.1.270

52 Bosisio, D. *et al.* Blocking TH17-polarizing cytokines by histone deacetylase inhibitors in vitro and in vivo. *J Leukoc Biol* **84**, 1540-1548 (2008). https://doi.org:10.1189/jlb.0708401

53 Dilek, N. *et al.* Control of transplant tolerance and intragraft regulatory T cell localization by myeloid-derived suppressor cells and CCL5. *J Immunol* **188**, 4209-4216 (2012). https://doi.org:10.4049/jimmunol.1101512

54 Laubli, H., Spanaus, K. S. & Borsig, L. Selectin-mediated activation of endothelial cells induces expression of CCL5 and promotes metastasis through recruitment of monocytes. *Blood* **114**, 4583-4591 (2009). https://doi.org:10.1182/blood-2008-10-186585

55 Hwaiz, R., Rahman, M., Syk, I., Zhang, E. & Thorlacius, H. Rac1-dependent secretion of platelet-derived CCL5 regulates neutrophil recruitment via activation of alveolar macrophages in septic lung injury. *J Leukoc Biol* **97**, 975-984 (2015). https://doi.org:10.1189/jlb.4A1214-603R

56 Zhang, Q. *et al.* CCL5-Mediated Th2 Immune Polarization Promotes Metastasis in Luminal Breast Cancer. *Cancer Res* **75**, 4312-4321 (2015). https://doi.org:10.1158/0008-5472.CAN-14-3590

57 Chen, M. *et al.* Identification of a novel biomarker-CCL5 using antibody microarray for colorectal cancer. *Pathol Res Pract* **215**, 1033-1037 (2019). https://doi.org:10.1016/j.prp.2019.02.011

58 Piryani, S. O., Kam, A. Y. F., Vu, U. T., Chao, N. J. & Doan, P. L. CCR5 Signaling Promotes Murine and Human Hematopoietic Regeneration following Ionizing Radiation. *Stem Cell Reports* **13**, 76-90 (2019). https://doi.org:10.1016/j.stemcr.2019.04.023

59 Ergen, A. V., Boles, N. C. & Goodell, M. A. Rantes/Ccl5 influences hematopoietic stem cell subtypes and causes myeloid skewing. *Blood* **119**, 2500-2509 (2012). https://doi.org:10.1182/blood-2011-11-391730

60 Astry, B. *et al.* Celastrol, a Chinese herbal compound, controls autoimmune inflammation by altering the balance of pathogenic and regulatory T cells in the target organ. *Clin Immunol* **157**, 228-238 (2015). https://doi.org:10.1016/j.clim.2015.01.011

61 Ponomarev, E. D. Fresh Evidence for Platelets as Neuronal and Innate Immune Cells: Their Role in the Activation, Differentiation, and Deactivation of Th1, Th17, and Tregs during Tissue Inflammation. *Front Immunol* **9**, 406 (2018). https://doi.org:10.3389/fimmu.2018.00406

62 Gouwy, M., Struyf, S., Proost, P. & Van Damme, J. Synergy in cytokine and chemokine networks amplifies the inflammatory response. *Cytokine Growth Factor Rev* **16**, 561-580 (2005). https://doi.org:10.1016/j.cytogfr.2005.03.005

63 Ford, J. *et al.* CCL7 Is a Negative Regulator of Cutaneous Inflammation Following Leishmania major Infection. *Front Immunol* **9**, 3063 (2018). https://doi.org:10.3389/fimmu.2018.03063

64 Szymczak, W. A. & Deepe, G. S., Jr. The CCL7-CCL2-CCR2 axis regulates IL-4 production in lungs and fungal immunity. *J Immunol* **183**, 1964-1974 (2009). https://doi.org:10.4049/jimmunol.0901316

65 Konno, K., Sasaki, T., Kulkeaw, K. & Sugiyama, D. Paracrine CCL17 and CCL22 signaling regulates hematopoietic stem/progenitor cell migration and retention in mouse fetal liver. *Biochem Biophys Res Commun* **527**, 730-736 (2020). https://doi.org:10.1016/j.bbrc.2020.04.045

66 Ruytinx, P., Proost, P., Van Damme, J. & Struyf, S. Chemokine-Induced Macrophage Polarization in Inflammatory Conditions. *Front Immunol* **9**, 1930 (2018). https://doi.org:10.3389/fimmu.2018.01930

67 Nakayama, T. *et al.* Selective induction of Th2-attracting chemokines CCL17 and CCL22 in human B cells by latent membrane protein 1 of Epstein-Barr virus. *J Virol* **78**, 1665-1674 (2004). https://doi.org:10.1128/jvi.78.4.1665-1674.2004

68 Yang, X. W. *et al.* Recruitment and significance of Th22 cells and Th17 cells in malignant ascites. *Oncol Lett* **16**, 5389-5397 (2018). https://doi.org:10.3892/ol.2018.9316

69 Kornerup, K. N., Salmon, G. P., Pitchford, S. C., Liu, W. L. & Page, C. P. Circulating platelet-neutrophil complexes are important for subsequent neutrophil activation and migration. *J Appl Physiol (1985)* **109**, 758-767 (2010). https://doi.org:10.1152/japplphysiol.01086.2009

70 Kimura, S. *et al.* Relationship between CCL22 Expression by Vascular Smooth Muscle Cells and Macrophage Histamine Receptors in Atherosclerosis. *J Atheroscler Thromb* **25**, 1240-1254 (2018). https://doi.org:10.5551/jat.44297

71 Ushio, A. *et al.* CCL22-Producing Resident Macrophages Enhance T Cell Response in Sjogren's Syndrome. *Front Immunol* **9**, 2594 (2018). https://doi.org:10.3389/fimmu.2018.02594
72 Pan, Y. *et al.* Neurotactin, a membrane-anchored chemokine upregulated in brain inflammation. *Nature* **387**, 611-617 (1997). https://doi.org:10.1038/42491
73 Suzuki, F. *et al.* Inhibition of CX3CL1 (fractalkine) improves experimental autoimmune myositis in SJL/J mice. *J Immunol* **175**, 6987-6996 (2005). https://doi.org:10.4049/jimmunol.175.10.6987
74 Fan, M. *et al.* Dihydroartemisinin derivative DC32 attenuates collagen-induced arthritis in mice by restoring the Treg/Th17 balance and inhibiting synovitis through down-regulation of IL-6. *Int Immunopharmacol* **65**, 233-243 (2018). https://doi.org:10.1016/j.intimp.2018.10.015
75 Aqmasheh, S., Shamsasanjan, K., Akbarzadehlaleh, P., Pashoutan Sarvar, D. & Timari, H. Effects of Mesenchymal Stem Cell Derivatives on Hematopoiesis and Hematopoietic Stem Cells. *Adv Pharm Bull* **7**, 165-177 (2017). https://doi.org:10.15171/apb.2017.021
76 Dong, L. *et al.* T Cell CX3CR1 Mediates Excess Atherosclerotic Inflammation in Renal Impairment. *J Am Soc Nephrol* **27**, 1753-1764 (2016). https://doi.org:10.1681/ASN.2015050540
77 Fu, D. *et al.* Fractalkine mediates lymphocyte inflammation and tubulointerstitial lesions by modifying the Treg/Th17 balance in lupus-prone MRL/lpr mice. *Am J Transl Res* **12**, 6170-6186 (2020).
78 Fraticelli, P. *et al.* Fractalkine (CX3CL1) as an amplification circuit of polarized Th1 responses. *J Clin Invest* **107**, 1173-1181 (2001). https://doi.org:10.1172/JCI11517
79 King, A. G. *et al.* Identification of unique truncated KC/GRO beta chemokines with potent hematopoietic and anti-infective activities. *J Immunol* **164**, 3774-3782 (2000). https://doi.org:10.4049/jimmunol.164.7.3774
80 Chintakuntlawar, A. V. & Chodosh, J. Chemokine CXCL1/KC and its receptor CXCR2 are responsible for neutrophil chemotaxis in adenoviral keratitis. *J Interferon Cytokine Res* **29**, 657-666 (2009). https://doi.org:10.1089/jir.2009.0006
81 Lv, M. *et al.* miR141-CXCL1-CXCR2 signaling-induced Treg recruitment regulates metastases and survival of non-small cell lung cancer. *Mol Cancer Ther* **13**, 3152-3162 (2014). https://doi.org:10.1158/1535-7163.MCT-14-0448
82 Jin, L., Batra, S., Douda, D. N., Palaniyar, N. & Jeyaseelan, S. CXCL1 contributes to host defense in polymicrobial sepsis via modulating T cell and neutrophil functions. *J Immunol* **193**, 3549-3558 (2014). https://doi.org:10.4049/jimmunol.1401138
83 Proost, P. *et al.* Human and bovine granulocyte chemotactic protein-2: complete amino acid sequence and functional characterization as chemokines. *Biochemistry* **32**, 10170-10177 (1993). https://doi.org:10.1021/bi00089a037
84 Hess, C. *et al.* IL-8 responsiveness defines a subset of CD8 T cells poised to kill. *Blood* **104**, 3463-3471 (2004). https://doi.org:10.1182/blood-2004-03-1067
85 Valent, P. & Dahinden, C. A. Role of interleukins in the regulation of basophil development and secretion. *Curr Opin Hematol* **17**, 60-66 (2010). https://doi.org:10.1097/MOH.0b013e328331fae9

86 Qin, L., Feng, J., Hu, C., Li, Y. & Niu, R. [Th17/Treg imbalance mediated by IL-8 in RSV-infected bronchial epithelial cells]. *Zhong Nan Da Xue Xue Bao Yi Xue Ban* **41**, 337-344 (2016). https://doi.org:10.11817/j.issn.1672-7347.2016.04.001

87 Gasch, M. *et al.* Generation of IL-8 and IL-9 producing CD4(+) T cells is affected by Th17 polarizing conditions and AHR ligands. *Mediators Inflamm* **2014**, 182549 (2014). https://doi.org:10.1155/2014/182549

88 Gandhi, R. *et al.* Cutting edge: human latency-associated peptide+ T cells: a novel regulatory T cell subset. *J Immunol* **184**, 4620-4624 (2010). https://doi.org:10.4049/jimmunol.0903329

89 Rotondi, M., Chiovato, L., Romagnani, S., Serio, M. & Romagnani, P. Role of chemokines in endocrine autoimmune diseases. *Endocr Rev* **28**, 492-520 (2007). https://doi.org:10.1210/er.2006-0044

90 Li, J. *et al.* Pro-inflammatory effects of the Th1 chemokine CXCL10 in acquired aplastic anaemia. *Cytokine* **94**, 45-51 (2017). https://doi.org:10.1016/j.cyto.2017.04.010

91 Lee, E. Y., Lee, Z. H. & Song, Y. W. CXCL10 and autoimmune diseases. *Autoimmun Rev* **8**, 379-383 (2009). https://doi.org:10.1016/j.autrev.2008.12.002

92 Nastase, M. V. *et al.* Biglycan, a novel trigger of Th1 and Th17 cell recruitment into the kidney. *Matrix Biol* **68-69**, 293-317 (2018). https://doi.org:10.1016/j.matbio.2017.12.002

93 Bosch-Camos, L. *et al.* Cross-protection against African swine fever virus upon intranasal vaccination is associated with an adaptive-innate immune crosstalk. *PLoS Pathog* **18**, e1010931 (2022). https://doi.org:10.1371/journal.ppat.1010931

94 Griffith, G. L. *et al.* CAP37 activation of PKC promotes human corneal epithelial cell chemotaxis. *Invest Ophthalmol Vis Sci* **54**, 6712-6723 (2013). https://doi.org:10.1167/iovs.13-12054

95 Nassif, P. S., Simpson, S. Q., Izzo, A. A. & Nicklaus, P. J. Epidermal growth factor and transforming growth factor-alpha in middle ear effusion. *Otolaryngol Head Neck Surg* **119**, 564-568 (1998). https://doi.org:10.1016/S0194-5998(98)70012-9

96 Kilroy, G. E. *et al.* Cytokine profile of human adipose-derived stem cells: expression of angiogenic, hematopoietic, and pro-inflammatory factors. *J Cell Physiol* **212**, 702-709 (2007). https://doi.org:10.1002/jcp.21068

97 Cai, M. *et al.* A Lectin-EGF antibody promotes regulatory T cells and attenuates nephrotoxic nephritis via DC-SIGN on dendritic cells. *J Transl Med* **11**, 103 (2013). https://doi.org:10.1186/1479-5876-11-103

98 Choi, B. D. *et al.* Systemic administration of a bispecific antibody targeting EGFRvIII successfully treats intracerebral glioma. *Proc Natl Acad Sci U S A* **110**, 270-275 (2013). https://doi.org:10.1073/pnas.1219817110

99 Torres-Castro, P. *et al.* Modulation of the Systemic Immune Response in Suckling Rats by Breast Milk TGF-beta2, EGF and FGF21 Supplementation. *Nutrients* **12** (2020). https://doi.org:10.3390/nu12061888

100 Kita, H. & Gleich, G. J. Chemokines active on eosinophils: potential roles in allergic inflammation. *J Exp Med* **183**, 2421-2426 (1996). https://doi.org:10.1084/jem.183.6.2421

101 Ponath, P. D. *et al.* Cloning of the human eosinophil chemoattractant, eotaxin. Expression, receptor binding, and functional properties suggest a mechanism for the selective recruitment of eosinophils. *J Clin Invest* **97**, 604-612 (1996). https://doi.org:10.1172/JCI118456

102 Ye, B. Q., Geng, Z. H., Ma, L. & Geng, J. G. Slit2 regulates attractive eosinophil and repulsive neutrophil chemotaxis through differential srGAP1 expression during lung inflammation. *J Immunol* **185**, 6294-6305 (2010). https://doi.org:10.4049/jimmunol.1001648

103 Rothenberg, M. E. *et al.* Eotaxin triggers eosinophil-selective chemotaxis and calcium flux via a distinct receptor and induces pulmonary eosinophilia in the presence of interleukin 5 in mice. *Mol Med* **2**, 334-348 (1996).

104 Quackenbush, E. J., Aguirre, V., Wershil, B. K. & Gutierrez-Ramos, J. C. Eotaxin influences the development of embryonic hematopoietic progenitors in the mouse. *J Leukoc Biol* **62**, 661-666 (1997). https://doi.org:10.1002/jlb.62.5.661

105 Rothenberg, M. E., MacLean, J. A., Pearlman, E., Luster, A. D. & Leder, P. Targeted disruption of the chemokine eotaxin partially reduces antigen-induced tissue eosinophilia. *J Exp Med* **185**, 785-790 (1997). https://doi.org:10.1084/jem.185.4.785

106 Antoine, M. *et al.* Fibroblast growth factor 16 and 18 are expressed in human cardiovascular tissues and induce on endothelial cells migration but not proliferation. *Biochem Biophys Res Commun* **346**, 224-233 (2006). https://doi.org:10.1016/j.bbrc.2006.05.105

107 Allouche, M. & Bikfalvi, A. The role of fibroblast growth factor-2 (FGF-2) in hematopoiesis. *Prog Growth Factor Res* **6**, 35-48 (1995). https://doi.org:10.1016/0955-2235(95)00041-0

108 Shao, X. *et al.* FGF2 cooperates with IL-17 to promote autoimmune inflammation. *Sci Rep* **7**, 7024 (2017). https://doi.org:10.1038/s41598-017-07597-8

109 Sitnicka, E. *et al.* Key role of flt3 ligand in regulation of the common lymphoid progenitor but not in maintenance of the hematopoietic stem cell pool. *Immunity* **17**, 463-472 (2002). https://doi.org:10.1016/s1074-7613(02)00419-3

110 Biswas, M. *et al.* Synergy between rapamycin and FLT3 ligand enhances plasmacytoid dendritic cell-dependent induction of CD4+CD25+FoxP3+ Treg. *Blood* **125**, 2937-2947 (2015). https://doi.org:10.1182/blood-2014-09-599266

111 Swee, L. K., Bosco, N., Malissen, B., Ceredig, R. & Rolink, A. Expansion of peripheral naturally occurring T regulatory cells by Fms-like tyrosine kinase 3 ligand treatment. *Blood* **113**, 6277-6287 (2009). https://doi.org:10.1182/blood-2008-06-161026

112 Numata, A. *et al.* Signal transducers and activators of transcription 3 augments the transcriptional activity of CCAAT/enhancer-binding protein alpha in granulocyte colony-stimulating factor signaling pathway. *J Biol Chem* **280**, 12621-12629 (2005). https://doi.org:10.1074/jbc.M408442200

113 Inoue, D. *et al.* Myelodysplastic syndromes are induced by histone methylation-altering ASXL1 mutations. *J Clin Invest* **123**, 4627-4640 (2013). https://doi.org:10.1172/JCI70739

114 Chee, L. C., Hendy, J., Purton, L. E. & McArthur, G. A. The granulocyte-colony stimulating factor receptor (G-CSFR) interacts with retinoic acid receptors (RARs) in the regulation of

myeloid differentiation. *J Leukoc Biol* **93**, 235-243 (2013). https://doi.org:10.1189/jlb.1211609

115 Brook, B. *et al.* BCG vaccination-induced emergency granulopoiesis provides rapid protection from neonatal sepsis. *Sci Transl Med* **12** (2020). https://doi.org:10.1126/scitranslmed.aax4517

116 Semerad, C. L., Poursine-Laurent, J., Liu, F. & Link, D. C. A role for G-CSF receptor signaling in the regulation of hematopoietic cell function but not lineage commitment or differentiation. *Immunity* **11**, 153-161 (1999). https://doi.org:10.1016/s1074-7613(00)80090-4

117 van Eck van der Sluijs, J. *et al.* Clinically applicable CD34(+)-derived blood dendritic cell subsets exhibit key subset-specific features and potently boost anti-tumor T and NK cell responses. *Cancer Immunol Immunother* **70**, 3167-3181 (2021). https://doi.org:10.1007/s00262-021-02899-3

118 Toh, H. C. *et al.* G-CSF induces a potentially tolerant gene and immunophenotype profile in T cells in vivo. *Clin Immunol* **132**, 83-92 (2009). https://doi.org:10.1016/j.clim.2009.03.509

119 Chen, J. *et al.* A new isoform of interleukin-3 receptor alpha with novel differentiation activity and high affinity binding mode. *J Biol Chem* **284**, 5763-5773 (2009). https://doi.org:10.1074/jbc.M808197200

120 Ujike, A. *et al.* Impaired dendritic cell maturation and increased T(H)2 responses in PIR-B(-/-) mice. *Nat Immunol* **3**, 542-548 (2002). https://doi.org:10.1038/ni801

121 Kared, H. *et al.* Role of GM-CSF in tolerance induction by mobilized hematopoietic progenitors. *Blood* **112**, 2575-2578 (2008). https://doi.org:10.1182/blood-2008-02-140681

122 McGeachy, M. J. GM-CSF: the secret weapon in the T(H)17 arsenal. *Nat Immunol* **12**, 521-522 (2011). https://doi.org:10.1038/ni.2044

123 Ko, H. J. *et al.* GM-CSF-responsive monocyte-derived dendritic cells are pivotal in Th17 pathogenesis. *J Immunol* **192**, 2202-2209 (2014). https://doi.org:10.4049/jimmunol.1302040

124 Schelenz, S., Smith, D. A. & Bancroft, G. J. Cytokine and chemokine responses following pulmonary challenge with Aspergillus fumigatus: obligatory role of TNF-alpha and GM-CSF in neutrophil recruitment. *Med Mycol* **37**, 183-194 (1999). https://doi.org:10.1046/j.1365-280x.1999.00219.x

125 Gomez-Cambronero, J. Rapamycin inhibits GM-CSF-induced neutrophil migration. *FEBS Lett* **550**, 94-100 (2003). https://doi.org:10.1016/s0014-5793(03)00828-7

126 Sheng, J. R., Muthusamy, T., Prabhakar, B. S. & Meriggioli, M. N. GM-CSF-induced regulatory T cells selectively inhibit anti-acetylcholine receptor-specific immune responses in experimental myasthenia gravis. *J Neuroimmunol* **240-241**, 65-73 (2011). https://doi.org:10.1016/j.jneuroim.2011.10.010

127 Boettcher, S. *et al.* Endothelial cells translate pathogen signals into G-CSF-driven emergency granulopoiesis. *Blood* **124**, 1393-1403 (2014). https://doi.org:10.1182/blood-2014-04-570762

128 Boettcher, S. & Manz, M. G. Sensing and translation of pathogen signals into demand-adapted myelopoiesis. *Curr Opin Hematol* **23**, 5-10 (2016). https://doi.org:10.1097/MOH.0000000000000201

129 Yoshimura, T. *et al.* Induction of Monocyte Chemoattractant Proteins in Macrophages via the Production of Granulocyte/Macrophage Colony-Stimulating Factor by Breast Cancer Cells. *Front Immunol* **7**, 2 (2016). https://doi.org:10.3389/fimmu.2016.00002

130 Hamilton, J. A. GM-CSF-Dependent Inflammatory Pathways. *Front Immunol* **10**, 2055 (2019). https://doi.org:10.3389/fimmu.2019.02055

131 Regan-Komito, D. *et al.* GM-CSF drives dysregulated hematopoietic stem cell activity and pathogenic extramedullary myelopoiesis in experimental spondyloarthritis. *Nat Commun* **11**, 155 (2020). https://doi.org:10.1038/s41467-019-13853-4

132 Passegue, E. & Ernst, P. IFN-alpha wakes up sleeping hematopoietic stem cells. *Nat Med* **15**, 612-613 (2009). https://doi.org:10.1038/nm0609-612

133 Moschen, A. R., Geiger, S., Krehan, I., Kaser, A. & Tilg, H. Interferon-alpha controls IL-17 expression in vitro and in vivo. *Immunobiology* **213**, 779-787 (2008). https://doi.org:10.1016/j.imbio.2008.07.022

134 Hirohata, S., Shibuya, H. & Tejima, S. Suppressive influences of IFN-alpha on IL-17 expression in human CD4+ T cells. *Clin Immunol* **134**, 340-344 (2010). https://doi.org:10.1016/j.clim.2009.11.012

135 Liu, X., Diedrichs-Mohring, M. & Wildner, G. The Role of IFN-alpha in Experimental and Clinical Uveitis. *Ocul Immunol Inflamm* **27**, 23-33 (2019). https://doi.org:10.1080/09273948.2017.1298822

136 Santini, S. M. *et al.* Interferon-alpha-conditioned human monocytes combine a Th1-orienting attitude with the induction of autologous Th17 responses: role of IL-23 and IL-12. *PLoS One* **6**, e17364 (2011). https://doi.org:10.1371/journal.pone.0017364

137 Riley, C. H. *et al.* Increase in circulating CD4(+)CD25(+)Foxp3(+) T cells in patients with Philadelphia-negative chronic myeloproliferative neoplasms during treatment with IFN-alpha. *Blood* **118**, 2170-2173 (2011). https://doi.org:10.1182/blood-2011-03-340992

138 McFarland, A. P. *et al.* Localized delivery of interferon-beta by Lactobacillus exacerbates experimental colitis. *PLoS One* **6**, e16967 (2011). https://doi.org:10.1371/journal.pone.0016967

139 Das, G., Sheridan, S. & Janeway, C. A., Jr. The source of early IFN-gamma that plays a role in Th1 priming. *J Immunol* **167**, 2004-2010 (2001). https://doi.org:10.4049/jimmunol.167.4.2004

140 Arora, P. *et al.* Body fluid from the parasitic worm Ascaris suum inhibits broad-acting pro-inflammatory programs in dendritic cells. *Immunology* **159**, 322-334 (2020). https://doi.org:10.1111/imm.13151

141 de Bruin, A. M. *et al.* IFNgamma induces monopoiesis and inhibits neutrophil development during inflammation. *Blood* **119**, 1543-1554 (2012). https://doi.org:10.1182/blood-2011-07-367706

142 McLoughlin, R. M. *et al.* Interplay between IFN-gamma and IL-6 signaling governs neutrophil trafficking and apoptosis during acute inflammation. *J Clin Invest* **112**, 598-607 (2003). https://doi.org:10.1172/JCI17129

143 Wood, K. J. & Sawitzki, B. Interferon gamma: a crucial role in the function of induced regulatory T cells in vivo. *Trends Immunol* **27**, 183-187 (2006). https://doi.org:10.1016/j.it.2006.02.008

144 Gajewski, T. F. & Fitch, F. W. Anti-proliferative effect of IFN-gamma in immune regulation. I. IFN-gamma inhibits the proliferation of Th2 but not Th1 murine helper T lymphocyte clones. *J Immunol* **140**, 4245-4252 (1988).

145 Corthay, A. How do regulatory T cells work? *Scand J Immunol* **70**, 326-336 (2009). https://doi.org:10.1111/j.1365-3083.2009.02308.x

146 Singh, R. P. *et al.* Th17 cells in inflammation and autoimmunity. *Autoimmun Rev* **13**, 1174-1181 (2014). https://doi.org:10.1016/j.autrev.2014.08.019

147 Mettelman, R. C., Allen, E. K. & Thomas, P. G. Mucosal immune responses to infection and vaccination in the respiratory tract. *Immunity* **55**, 749-780 (2022). https://doi.org:10.1016/j.immuni.2022.04.013

148 Elser, B. *et al.* IFN-gamma represses IL-4 expression via IRF-1 and IRF-2. *Immunity* **17**, 703-712 (2002). https://doi.org:10.1016/s1074-7613(02)00471-5

149 Von Stebut, E. *et al.* Interleukin 1alpha promotes Th1 differentiation and inhibits disease progression in Leishmania major-susceptible BALB/c mice. *J Exp Med* **198**, 191-199 (2003). https://doi.org:10.1084/jem.20030159

150 Lamacchia, C., Palmer, G., Seemayer, C. A., Talabot-Ayer, D. & Gabay, C. Enhanced Th1 and Th17 responses and arthritis severity in mice with a deficiency of myeloid cell-specific interleukin-1 receptor antagonist. *Arthritis Rheum* **62**, 452-462 (2010). https://doi.org:10.1002/art.27235

151 Santarlasci, V., Cosmi, L., Maggi, L., Liotta, F. & Annunziato, F. IL-1 and T Helper Immune Responses. *Front Immunol* **4**, 182 (2013). https://doi.org:10.3389/fimmu.2013.00182

152 Johnson, C. S., Keckler, D. J., Topper, M. I., Braunschweiger, P. G. & Furmanski, P. In vivo hematopoietic effects of recombinant interleukin-1 alpha in mice: stimulation of granulocytic, monocytic, megakaryocytic, and early erythroid progenitors, suppression of late-stage erythropoiesis, and reversal of erythroid suppression with erythropoietin. *Blood* **73**, 678-683 (1989).

153 Ketelut-Carneiro, N. *et al.* Caspase-11-dependent IL-1alpha release boosts Th17 immunity against Paracoccidioides brasiliensis. *PLoS Pathog* **15**, e1007990 (2019). https://doi.org:10.1371/journal.ppat.1007990

154 Manzanillo, P., Eidenschenk, C. & Ouyang, W. Deciphering the crosstalk among IL-1 and IL-10 family cytokines in intestinal immunity. *Trends Immunol* **36**, 471-478 (2015). https://doi.org:10.1016/j.it.2015.06.003

155 Carmi, Y. *et al.* Microenvironment-derived IL-1 and IL-17 interact in the control of lung metastasis. *J Immunol* **186**, 3462-3471 (2011). https://doi.org:10.4049/jimmunol.1002901

156 Jeffery, H. C. *et al.* Bidirectional Cross-Talk between Biliary Epithelium and Th17 Cells Promotes Local Th17 Expansion and Bile Duct Proliferation in Biliary Liver Diseases. *J Immunol* **203**, 1151-1159 (2019). https://doi.org:10.4049/jimmunol.1800455

157 Zhao, Y. *et al.* Shifted T Helper Cell Polarization in a Murine Staphylococcus aureus Mastitis Model. *PLoS One* **10**, e0134797 (2015). https://doi.org:10.1371/journal.pone.0134797

158 Bigildeev, A. E., Zezina, E. A., Shipounova, I. N. & Drize, N. J. Interleukin-1 beta enhances human multipotent mesenchymal stromal cell proliferative potential and their ability to maintain hematopoietic precursor cells. *Cytokine* **71**, 246-254 (2015). https://doi.org:10.1016/j.cyto.2014.10.018

159 Kobayashi, M. *et al.* Synergistic effects of interleukin-1 beta and interleukin-3 on the expansion of human hematopoietic progenitor cells in liquid cultures. *Blood* **78**, 1947-1953 (1991).

160 Iizumi, T. *et al.* Recombinant human interleukin-1 beta analogue as a regulator of hematopoiesis in patients receiving chemotherapy for urogenital cancers. *Cancer* **68**, 1520-1523 (1991). https://doi.org:10.1002/1097-0142(19911001)68:7<1520::aid-cncr2820680710>3.0.co;2-2

161 Imamura, M. *et al.* In vitro expansion of murine hematopoietic progenitor cells by leukemia inhibitory factor, stem cell factor, and interleukin-1 beta. *Exp Hematol* **24**, 1280-1288 (1996).

162 Shourian, M., Ralph, B., Angers, I., Sheppard, D. C. & Qureshi, S. T. Contribution of IL-1RI Signaling to Protection against Cryptococcus neoformans 52D in a Mouse Model of Infection. *Front Immunol* **8**, 1987 (2017). https://doi.org:10.3389/fimmu.2017.01987

163 Arts, R. J. W. *et al.* BCG Vaccination Protects against Experimental Viral Infection in Humans through the Induction of Cytokines Associated with Trained Immunity. *Cell Host Microbe* **23**, 89-100 e105 (2018). https://doi.org:10.1016/j.chom.2017.12.010

164 Ciarlo, E. *et al.* Trained Immunity Confers Broad-Spectrum Protection Against Bacterial Infections. *J Infect Dis* **222**, 1869-1881 (2020). https://doi.org:10.1093/infdis/jiz692

165 Murugaiyan, G. *et al.* MicroRNA-21 promotes Th17 differentiation and mediates experimental autoimmune encephalomyelitis. *J Clin Invest* **125**, 1069-1080 (2015). https://doi.org:10.1172/JCI74347

166 Swain, S. L. T-cell subsets. Who does the polarizing? *Curr Biol* **5**, 849-851 (1995). https://doi.org:10.1016/s0960-9822(95)00170-9

167 Spellberg, B. & Edwards, J. E., Jr. Type 1/Type 2 immunity in infectious diseases. *Clin Infect Dis* **32**, 76-102 (2001). https://doi.org:10.1086/317537

168 Pandit, M. *et al.* Geranylgeranyl pyrophosphate amplifies T(reg) differentiation via increased IL-2 expression to ameliorate DSS-induced colitis. *Eur J Immunol* **51**, 1461-1472 (2021). https://doi.org:10.1002/eji.202048991

169 Rocha, A. M. *et al.* IL1RN VNTR and IL2-330 polymorphic genes are independently associated with chronic immune thrombocytopenia. *Br J Haematol* **150**, 679-684 (2010). https://doi.org:10.1111/j.1365-2141.2010.08318.x

170 Noble, A., Thomas, M. J. & Kemeny, D. M. Early Th1/Th2 cell polarization in the absence of IL-4 and IL-12: T cell receptor signaling regulates the response to cytokines in CD4 and CD8 T cells. *Eur J Immunol* **31**, 2227-2235 (2001). https://doi.org:10.1002/1521-4141(200107)31:7<2227::aid-immu2227>3.0.co;2-c

171 Boyman, O. & Sprent, J. The role of interleukin-2 during homeostasis and activation of the immune system. *Nat Rev Immunol* **12**, 180-190 (2012). https://doi.org:10.1038/nri3156

172 Luo, J. *et al.* IL-2 Inhibition of Th17 Generation Rather Than Induction of Treg Cells Is Impaired in Primary Sjogren's Syndrome Patients. *Front Immunol* **9**, 1755 (2018). https://doi.org:10.3389/fimmu.2018.01755

173 Liao, W., Lin, J. X. & Leonard, W. J. Interleukin-2 at the crossroads of effector responses, tolerance, and immunotherapy. *Immunity* **38**, 13-25 (2013). https://doi.org:10.1016/j.immuni.2013.01.004

174 Tsuji, K., Lyman, S. D., Sudo, T., Clark, S. C. & Ogawa, M. Enhancement of murine hematopoiesis by synergistic interactions between steel factor (ligand for c-kit), interleukin-11, and other early acting factors in culture. *Blood* **79**, 2855-2860 (1992).

175 Ploemacher, R. E., van Soest, P. L., Boudewijn, A. & Neben, S. Interleukin-12 enhances interleukin-3 dependent multilineage hematopoietic colony formation stimulated by interleukin-11 or steel factor. *Leukemia* **7**, 1374-1380 (1993).

176 Kumar, A. *et al.* IL-3 Receptor Expression on Activated Human Th Cells Is Regulated by IL-4, and IL-3 Synergizes with IL-4 to Enhance Th2 Cell Differentiation. *J Immunol* **204**, 819-831 (2020). https://doi.org:10.4049/jimmunol.1801629

177 Manz, M. G. & Boettcher, S. Emergency granulopoiesis. *Nat Rev Immunol* **14**, 302-314 (2014). https://doi.org:10.1038/nri3660

178 Borriello, F. *et al.* GM-CSF and IL-3 Modulate Human Monocyte TNF-alpha Production and Renewal in In Vitro Models of Trained Immunity. *Front Immunol* **7**, 680 (2016). https://doi.org:10.3389/fimmu.2016.00680

179 Stockinger, B. & Veldhoen, M. Differentiation and function of Th17 T cells. *Curr Opin Immunol* **19**, 281-286 (2007). https://doi.org:10.1016/j.coi.2007.04.005

180 Chen, L. *et al.* IL-4 induces differentiation and expansion of Th2 cytokine-producing eosinophils. *J Immunol* **172**, 2059-2066 (2004). https://doi.org:10.4049/jimmunol.172.4.2059

181 Darlan, D. M., Rozi, M. F. & Yulfi, H. Overview of Immunological Responses and Immunomodulation Properties of Trichuris sp.: Prospects for Better Understanding Human Trichuriasis. *Life (Basel)* **11** (2021). https://doi.org:10.3390/life11030188

182 Harrington, L. E. *et al.* Interleukin 17-producing CD4+ effector T cells develop via a lineage distinct from the T helper type 1 and 2 lineages. *Nat Immunol* **6**, 1123-1132 (2005). https://doi.org:10.1038/ni1254

183 van Hamburg, J. P. *et al.* Enforced expression of GATA3 allows differentiation of IL-17-producing cells, but constrains Th17-mediated pathology. *Eur J Immunol* **38**, 2573-2586 (2008). https://doi.org:10.1002/eji.200737840

184 Keller, U., Aman, M. J., Derigs, G., Huber, C. & Peschel, C. Human interleukin-4 enhances stromal cell-dependent hematopoiesis: costimulation with stem cell factor. *Blood* **84**, 2189-2196 (1994).

185 Sonoda, Y. Interleukin-4--a dual regulatory factor in hematopoiesis. *Leuk Lymphoma* **14**, 231-240 (1994). https://doi.org:10.3109/10428199409049673

186 Bonecchi, R. *et al.* Divergent effects of interleukin-4 and interferon-gamma on macrophage-derived chemokine production: an amplification circuit of polarized T helper 2 responses. *Blood* **92**, 2668-2671 (1998).

187 Munitz, A., Brandt, E. B., Mingler, M., Finkelman, F. D. & Rothenberg, M. E. Distinct roles for IL-13 and IL-4 via IL-13 receptor alpha1 and the type II IL-4 receptor in asthma

pathogenesis. *Proc Natl Acad Sci U S A* **105**, 7240-7245 (2008). https://doi.org:10.1073/pnas.0802465105
188 Kagami, S. *et al.* Interleukin-4 and interleukin-13 enhance CCL26 production in a human keratinocyte cell line, HaCaT cells. *Clin Exp Immunol* **141**, 459-466 (2005). https://doi.org:10.1111/j.1365-2249.2005.02875.x
189 Jinquan, T. *et al.* CXC chemokine receptor 4 expression and stromal cell-derived factor-1alpha-induced chemotaxis in CD4+ T lymphocytes are regulated by interleukin-4 and interleukin-10. *Immunology* **99**, 402-410 (2000). https://doi.org:10.1046/j.1365-2567.2000.00954.x
190 Cousin, C. *et al.* The immunosuppressive enzyme IL4I1 promotes FoxP3(+) regulatory T lymphocyte differentiation. *Eur J Immunol* **45**, 1772-1782 (2015). https://doi.org:10.1002/eji.201445000
191 Baraut, J., Michel, L., Verrecchia, F. & Farge, D. Relationship between cytokine profiles and clinical outcomes in patients with systemic sclerosis. *Autoimmun Rev* **10**, 65-73 (2010). https://doi.org:10.1016/j.autrev.2010.08.003
192 Zhu, Y. *et al.* Cutting edge: IL-5 primes Th2 cytokine-producing capacity in eosinophils through a STAT5-dependent mechanism. *J Immunol* **173**, 2918-2922 (2004). https://doi.org:10.4049/jimmunol.173.5.2918
193 Ye, D. *et al.* Interleukin-5 levels are decreased in the plasma of coronary artery disease patients and inhibit Th1 and Th17 differentiation in vitro. *Rev Esp Cardiol (Engl Ed)* **73**, 393-402 (2020). https://doi.org:10.1016/j.rec.2019.07.005
194 Karnowski, A. *et al.* B and T cells collaborate in antiviral responses via IL-6, IL-21, and transcriptional activator and coactivator, Oct2 and OBF-1. *J Exp Med* **209**, 2049-2064 (2012). https://doi.org:10.1084/jem.20111504
195 Kusam, S., Toney, L. M., Sato, H. & Dent, A. L. Inhibition of Th2 differentiation and GATA-3 expression by BCL-6. *J Immunol* **170**, 2435-2441 (2003). https://doi.org:10.4049/jimmunol.170.5.2435
196 Heink, S. *et al.* Trans-presentation of IL-6 by dendritic cells is required for the priming of pathogenic T(H)17 cells. *Nat Immunol* **18**, 74-85 (2017). https://doi.org:10.1038/ni.3632
197 Ivanov, II *et al.* The orphan nuclear receptor RORgammat directs the differentiation program of proinflammatory IL-17+ T helper cells. *Cell* **126**, 1121-1133 (2006). https://doi.org:10.1016/j.cell.2006.07.035
198 Kimura, A. & Kishimoto, T. IL-6: regulator of Treg/Th17 balance. *Eur J Immunol* **40**, 1830-1835 (2010). https://doi.org:10.1002/eji.201040391
199 Blin-Wakkach, C., Wakkach, A., Quincey, D. & Carle, G. F. Interleukin-7 partially rescues B-lymphopoiesis in osteopetrotic oc/oc mice through the engagement of B220+ CD11b+ progenitors. *Exp Hematol* **34**, 851-859 (2006). https://doi.org:10.1016/j.exphem.2006.04.003
200 Datta, S. R. *et al.* Survival factor-mediated BAD phosphorylation raises the mitochondrial threshold for apoptosis. *Dev Cell* **3**, 631-643 (2002). https://doi.org:10.1016/s1534-5807(02)00326-x
201 Maraskovsky, E. *et al.* Bcl-2 can rescue T lymphocyte development in interleukin-7 receptor-deficient mice but not in mutant rag-1-/- mice. *Cell* **89**, 1011-1019 (1997). https://doi.org:10.1016/s0092-8674(00)80289-5

202 Lee, L. F. *et al.* IL-7 promotes T(H)1 development and serum IL-7 predicts clinical response to interferon-beta in multiple sclerosis. *Sci Transl Med* **3**, 93ra68 (2011). https://doi.org:10.1126/scitranslmed.3002400

203 van Roon, J. A., Glaudemans, C. A., Bijlsma, J. W. & Lafeber, F. P. Differentiation of naive CD4+ T cells towards T helper 2 cells is not impaired in rheumatoid arthritis patients. *Arthritis Res Ther* **5**, R269-276 (2003). https://doi.org:10.1186/ar790

204 Chen, X. *et al.* Thymic regulation of autoimmune disease by accelerated differentiation of Foxp3+ regulatory T cells through IL-7 signaling pathway. *J Immunol* **183**, 6135-6144 (2009). https://doi.org:10.4049/jimmunol.0901576

205 Lee, W. W. *et al.* Regulating human Th17 cells via differential expression of IL-1 receptor. *Blood* **115**, 530-540 (2010). https://doi.org:10.1182/blood-2009-08-236521

206 Chen, Y., Chauhan, S. K., Tan, X. & Dana, R. Interleukin-7 and -15 maintain pathogenic memory Th17 cells in autoimmunity. *J Autoimmun* **77**, 96-103 (2017). https://doi.org:10.1016/j.jaut.2016.11.003

207 Do-Thi, V. A., Lee, J. O., Lee, H. & Kim, Y. S. Crosstalk between the Producers and Immune Targets of IL-9. *Immune Netw* **20**, e45 (2020). https://doi.org:10.4110/in.2020.20.e45

208 Takatsuka, S. *et al.* IL-9 receptor signaling in memory B cells regulates humoral recall responses. *Nat Immunol* **19**, 1025-1034 (2018). https://doi.org:10.1038/s41590-018-0177-0

209 Yang, Y. C. Human interleukin-9: a new cytokine in hematopoiesis. *Leuk Lymphoma* **8**, 441-447 (1992). https://doi.org:10.3109/10428199209051026

210 Li, H. & Rostami, A. IL-9: basic biology, signaling pathways in CD4+ T cells and implications for autoimmunity. *J Neuroimmune Pharmacol* **5**, 198-209 (2010). https://doi.org:10.1007/s11481-009-9186-y

211 Elyaman, W. *et al.* Notch receptors and Smad3 signaling cooperate in the induction of interleukin-9-producing T cells. *Immunity* **36**, 623-634 (2012). https://doi.org:10.1016/j.immuni.2012.01.020

212 Elyaman, W. *et al.* IL-9 induces differentiation of TH17 cells and enhances function of FoxP3+ natural regulatory T cells. *Proc Natl Acad Sci U S A* **106**, 12885-12890 (2009). https://doi.org:10.1073/pnas.0812530106

213 Beriou, G. *et al.* TGF-beta induces IL-9 production from human Th17 cells. *J Immunol* **185**, 46-54 (2010). https://doi.org:10.4049/jimmunol.1000356

214 Gu, Z. W., Wang, Y. X. & Cao, Z. W. Neutralization of interleukin-9 ameliorates symptoms of allergic rhinitis by reducing Th2, Th9, and Th17 responses and increasing the Treg response in a murine model. *Oncotarget* **8**, 14314-14324 (2017). https://doi.org:10.18632/oncotarget.15177

215 Ye, Z. J. *et al.* Differentiation and immune regulation of IL-9-producing CD4+ T cells in malignant pleural effusion. *Am J Respir Crit Care Med* **186**, 1168-1179 (2012). https://doi.org:10.1164/rccm.201207-1307OC

216 Fang, D. & Zhu, J. Molecular switches for regulating the differentiation of inflammatory and IL-10-producing anti-inflammatory T-helper cells. *Cell Mol Life Sci* **77**, 289-303 (2020). https://doi.org:10.1007/s00018-019-03277-0

217 Couper, K. N., Blount, D. G. & Riley, E. M. IL-10: the master regulator of immunity to infection. *J Immunol* **180**, 5771-5777 (2008). https://doi.org:10.4049/jimmunol.180.9.5771

218 Ghannam, S., Pene, J., Moquet-Torcy, G., Jorgensen, C. & Yssel, H. Mesenchymal stem cells inhibit human Th17 cell differentiation and function and induce a T regulatory cell phenotype. *J Immunol* **185**, 302-312 (2010). https://doi.org:10.4049/jimmunol.0902007

219 Guo, B. IL-10 Modulates Th17 Pathogenicity during Autoimmune Diseases. *J Clin Cell Immunol* **7** (2016). https://doi.org:10.4172/2155-9899.1000400

220 Camacho, V. *et al.* Bone marrow Tregs mediate stromal cell function and support hematopoiesis via IL-10. *JCI Insight* **5** (2020). https://doi.org:10.1172/jci.insight.135681

221 Orgun, N. N., Mathis, M. A., Wilson, C. B. & Way, S. S. Deviation from a strong Th1-dominated to a modest Th17-dominated CD4 T cell response in the absence of IL-12p40 and type I IFNs sustains protective CD8 T cells. *J Immunol* **180**, 4109-4115 (2008). https://doi.org:10.4049/jimmunol.180.6.4109

222 Decken, K. *et al.* Interleukin-12 is essential for a protective Th1 response in mice infected with Cryptococcus neoformans. *Infect Immun* **66**, 4994-5000 (1998). https://doi.org:10.1128/IAI.66.10.4994-5000.1998

223 Lim, H. X., Hong, H. J., Jung, M. Y., Cho, D. & Kim, T. S. Principal role of IL-12p40 in the decreased Th1 and Th17 responses driven by dendritic cells of mice lacking IL-12 and IL-18. *Cytokine* **63**, 179-186 (2013). https://doi.org:10.1016/j.cyto.2013.04.029

224 Marks, E. *et al.* Regulation of IL-12p40 by HIF controls Th1/Th17 responses to prevent mucosal inflammation. *Mucosal Immunol* **10**, 1224-1236 (2017). https://doi.org:10.1038/mi.2016.135

225 Nigg, A. P. *et al.* Dendritic cell-derived IL-12p40 homodimer contributes to susceptibility in cutaneous leishmaniasis in BALB/c mice. *J Immunol* **178**, 7251-7258 (2007). https://doi.org:10.4049/jimmunol.178.11.7251

226 Drohomyrecky, P. C. *et al.* Peroxisome Proliferator-Activated Receptor-delta Acts within Peripheral Myeloid Cells to Limit Th Cell Priming during Experimental Autoimmune Encephalomyelitis. *J Immunol* **203**, 2588-2601 (2019). https://doi.org:10.4049/jimmunol.1801200

227 Brahmachari, S. & Pahan, K. Suppression of regulatory T cells by IL-12p40 homodimer via nitric oxide. *J Immunol* **183**, 2045-2058 (2009). https://doi.org:10.4049/jimmunol.0800276

228 Kubin, M., Kamoun, M. & Trinchieri, G. Interleukin 12 synergizes with B7/CD28 interaction in inducing efficient proliferation and cytokine production of human T cells. *J Exp Med* **180**, 211-222 (1994). https://doi.org:10.1084/jem.180.1.211

229 Wang, Q. *et al.* Dendritic cells support hematopoiesis of bone marrow cells. *Transplantation* **72**, 891-899 (2001). https://doi.org:10.1097/00007890-200109150-00026

230 Jacobsen, S. E. *et al.* Interleukin 13: novel role in direct regulation of proliferation and differentiation of primitive hematopoietic progenitor cells. *J Exp Med* **180**, 75-82 (1994). https://doi.org:10.1084/jem.180.1.75

231 Van Acker, H. H. *et al.* Interleukin-15 enhances the proliferation, stimulatory phenotype, and antitumor effector functions of human gamma delta T cells. *J Hematol Oncol* **9**, 101 (2016). https://doi.org:10.1186/s13045-016-0329-3

232 Kawamura, T., Koka, R., Ma, A. & Kumar, V. Differential roles for IL-15R alpha-chain in NK cell development and Ly-49 induction. *J Immunol* **171**, 5085-5090 (2003). https://doi.org:10.4049/jimmunol.171.10.5085

233 Ivanova, A. V. *et al.* Autoimmunity, spontaneous tumourigenesis, and IL-15 insufficiency in mice with a targeted disruption of the tumour suppressor gene Fus1. *J Pathol* **211**, 591-601 (2007). https://doi.org:10.1002/path.2146

234 Kennedy, M. K. *et al.* Reversible defects in natural killer and memory CD8 T cell lineages in interleukin 15-deficient mice. *J Exp Med* **191**, 771-780 (2000). https://doi.org:10.1084/jem.191.5.771

235 Niedbala, W., Wei, X. & Liew, F. Y. IL-15 induces type 1 and type 2 CD4+ and CD8+ T cells proliferation but is unable to drive cytokine production in the absence of TCR activation or IL-12 / IL-4 stimulation in vitro. *Eur J Immunol* **32**, 341-347 (2002). https://doi.org:10.1002/1521-4141(200202)32:2<341::AID-IMMU341>3.0.CO;2-X

236 Mahallawi, W. H., Khabour, O. F., Zhang, Q., Makhdoum, H. M. & Suliman, B. A. MERS-CoV infection in humans is associated with a pro-inflammatory Th1 and Th17 cytokine profile. *Cytokine* **104**, 8-13 (2018). https://doi.org:10.1016/j.cyto.2018.01.025

237 Ho, A. W. *et al.* IL-17RC is required for immune signaling via an extended SEF/IL-17R signaling domain in the cytoplasmic tail. *J Immunol* **185**, 1063-1070 (2010). https://doi.org:10.4049/jimmunol.0903739

238 Liang, S. C. *et al.* An IL-17F/A heterodimer protein is produced by mouse Th17 cells and induces airway neutrophil recruitment. *J Immunol* **179**, 7791-7799 (2007). https://doi.org:10.4049/jimmunol.179.11.7791

239 Awasthi, A. & Kuchroo, V. K. IL-17A directly inhibits TH1 cells and thereby suppresses development of intestinal inflammation. *Nat Immunol* **10**, 568-570 (2009). https://doi.org:10.1038/ni0609-568

240 Nakajima, S. *et al.* IL-17A as an inducer for Th2 immune responses in murine atopic dermatitis models. *J Invest Dermatol* **134**, 2122-2130 (2014). https://doi.org:10.1038/jid.2014.51

241 Mojsilovic, S., Jaukovic, A., Santibanez, J. F. & Bugarski, D. Interleukin-17 and its implication in the regulation of differentiation and function of hematopoietic and mesenchymal stem cells. *Mediators Inflamm* **2015**, 470458 (2015). https://doi.org:10.1155/2015/470458

242 Lee, S. Y. *et al.* IL-1 receptor antagonist (IL-1Ra)-Fc ameliorate autoimmune arthritis by regulation of the Th17 cells/Treg balance and arthrogenic cytokine activation. *Immunol Lett* **172**, 56-66 (2016). https://doi.org:10.1016/j.imlet.2016.02.011

243 Niu, X. *et al.* Regulatory immune responses induced by IL-1 receptor antagonist in rheumatoid arthritis. *Mol Immunol* **49**, 290-296 (2011). https://doi.org:10.1016/j.molimm.2011.08.020

244 Sugawara, R. *et al.* Small intestinal eosinophils regulate Th17 cells by producing IL-1 receptor antagonist. *J Exp Med* **213**, 555-567 (2016). https://doi.org:10.1084/jem.20141388

245 Schneider, L. *et al.* Directional cell migration and chemotaxis in wound healing response to PDGF-AA are coordinated by the primary cilium in fibroblasts. *Cell Physiol Biochem* **25**, 279-292 (2010). https://doi.org:10.1159/000276562

246 Osornio-Vargas, A. R. *et al.* Platelet-derived growth factor (PDGF)-AA, -AB, and -BB induce differential chemotaxis of early-passage rat lung fibroblasts in vitro. *Am J Respir Cell Mol Biol* **12**, 33-40 (1995). https://doi.org:10.1165/ajrcmb.12.1.7811469

247 Shure, D., Senior, R. M., Griffin, G. L. & Deuel, T. F. PDGF AA homodimers are potent chemoattractants for fibroblasts and neutrophils, and for monocytes activated by lymphocytes or cytokines. *Biochem Biophys Res Commun* **186**, 1510-1514 (1992). https://doi.org:10.1016/s0006-291x(05)81577-3

248 Heldin, C. H., Ostman, A. & Ronnstrand, L. Signal transduction via platelet-derived growth factor receptors. *Biochim Biophys Acta* **1378**, F79-113 (1998). https://doi.org:10.1016/s0304-419x(98)00015-8

249 Siegbahn, A., Hammacher, A., Westermark, B. & Heldin, C. H. Differential effects of the various isoforms of platelet-derived growth factor on chemotaxis of fibroblasts, monocytes, and granulocytes. *J Clin Invest* **85**, 916-920 (1990). https://doi.org:10.1172/JCI114519

250 Heldin, C. H. & Westermark, B. Mechanism of action and in vivo role of platelet-derived growth factor. *Physiol Rev* **79**, 1283-1316 (1999). https://doi.org:10.1152/physrev.1999.79.4.1283

251 Xue, Y. *et al.* PDGF-BB modulates hematopoiesis and tumor angiogenesis by inducing erythropoietin production in stromal cells. *Nat Med* **18**, 100-110 (2011). https://doi.org:10.1038/nm.2575

252 Kaminski, W. E. *et al.* Basis of hematopoietic defects in platelet-derived growth factor (PDGF)-B and PDGF beta-receptor null mice. *Blood* **97**, 1990-1998 (2001). https://doi.org:10.1182/blood.v97.7.1990

253 Koyama, N., Morisaki, N., Saito, Y. & Yoshida, S. Regulatory effects of platelet-derived growth factor-AA homodimer on migration of vascular smooth muscle cells. *J Biol Chem* **267**, 22806-22812 (1992).

254 Gomez, C. E., Najera, J. L., Sanchez, R., Jimenez, V. & Esteban, M. Multimeric soluble CD40 ligand (sCD40L) efficiently enhances HIV specific cellular immune responses during DNA prime and boost with attenuated poxvirus vectors MVA and NYVAC expressing HIV antigens. *Vaccine* **27**, 3165-3174 (2009). https://doi.org:10.1016/j.vaccine.2009.03.049

255 Zhang, Z. M. *et al.* Antitumor effects and mechanisms of dendritic cells stimulated by sCD40L on ovarian cancer cells in vitro. *Onco Targets Ther* **6**, 503-515 (2013). https://doi.org:10.2147/OTT.S40504

256 Damas, J. K. *et al.* Soluble CD40 ligand in pulmonary arterial hypertension: possible pathogenic role of the interaction between platelets and endothelial cells. *Circulation* **110**, 999-1005 (2004). https://doi.org:10.1161/01.CIR.0000139859.68513.FC

257 Nagahama, M. *et al.* Significance of chemokines and soluble CD40 ligand in patients with autoimmune thrombocytopenic purpura. *Eur J Haematol* **69**, 303-308 (2002). https://doi.org:10.1034/j.1600-0609.2002.02774.x

258 Lievens, D. *et al.* Platelet CD40L mediates thrombotic and inflammatory processes in atherosclerosis. *Blood* **116**, 4317-4327 (2010). https://doi.org:10.1182/blood-2010-01-261206

259 Dai, J. *et al.* Metabolism-associated danger signal-induced immune response and reverse immune checkpoint-activated CD40(+) monocyte differentiation. *J Hematol Oncol* **10**, 141 (2017). https://doi.org:10.1186/s13045-017-0504-1

260 Lopez-Abente, J., Correa-Rocha, R. & Pion, M. Functional Mechanisms of Treg in the Context of HIV Infection and the Janus Face of Immune Suppression. *Front Immunol* **7**, 192 (2016). https://doi.org:10.3389/fimmu.2016.00192

261 Jenabian, M. A. *et al.* Soluble CD40-ligand (sCD40L, sCD154) plays an immunosuppressive role via regulatory T cell expansion in HIV infection. *Clin Exp Immunol* **178**, 102-111 (2014). https://doi.org:10.1111/cei.12396

262 Zamora, C. *et al.* Binding of Platelets to Lymphocytes: A Potential Anti-Inflammatory Therapy in Rheumatoid Arthritis. *J Immunol* **198**, 3099-3108 (2017). https://doi.org:10.4049/jimmunol.1601708

263 Hasbold, J., Corcoran, L. M., Tarlinton, D. M., Tangye, S. G. & Hodgkin, P. D. Evidence from the generation of immunoglobulin G-secreting cells that stochastic mechanisms regulate lymphocyte differentiation. *Nat Immunol* **5**, 55-63 (2004). https://doi.org:10.1038/ni1016

264 Flores-Romo, L. *et al.* CD40 ligation on human cord blood CD34+ hematopoietic progenitors induces their proliferation and differentiation into functional dendritic cells. *J Exp Med* **185**, 341-349 (1997). https://doi.org:10.1084/jem.185.2.341

265 Franca, T. T. *et al.* CD40 ligand deficiency: treatment strategies and novel therapeutic perspectives. *Expert Rev Clin Immunol* **15**, 529-540 (2019). https://doi.org:10.1080/1744666X.2019.1573674

266 Masuda, H. *et al.* Soluble CD40 ligand disrupts the blood-brain barrier and exacerbates inflammation in experimental autoimmune encephalomyelitis. *J Neuroimmunol* **316**, 117-120 (2018). https://doi.org:10.1016/j.jneuroim.2018.01.001

267 Eriksson, U. *et al.* Interleukin-6-deficient mice resist development of autoimmune myocarditis associated with impaired upregulation of complement C3. *Circulation* **107**, 320-325 (2003). https://doi.org:10.1161/01.cir.0000043802.38699.66

268 Upparahalli Venkateshaiah, S. *et al.* Attenuation of Allergen-, IL-13-, and TGF-alpha-induced Lung Fibrosis after the Treatment of rIL-15 in Mice. *Am J Respir Cell Mol Biol* **61**, 97-109 (2019). https://doi.org:10.1165/rcmb.2018-0254OC

269 Pardanaud, L. & Dieterlen-Lievre, F. Manipulation of the angiopoietic/hemangiopoietic commitment in the avian embryo. *Development* **126**, 617-627 (1999). https://doi.org:10.1242/dev.126.4.617

270 Maciejewski, J. P. *et al.* Nitric oxide suppression of human hematopoiesis in vitro. Contribution to inhibitory action of interferon-gamma and tumor necrosis factor-alpha. *J Clin Invest* **96**, 1085-1092 (1995). https://doi.org:10.1172/JCI118094

271 Angelone, D. F. *et al.* Innate immunity of the human newborn is polarized toward a high ratio of IL-6/TNF-alpha production in vitro and in vivo. *Pediatr Res* **60**, 205-209 (2006). https://doi.org:10.1203/01.pdr.0000228319.10481.ea

272 Iwamoto, S. *et al.* TNF-alpha drives human CD14+ monocytes to differentiate into CD70+ dendritic cells evoking Th1 and Th17 responses. *J Immunol* **179**, 1449-1457 (2007). https://doi.org:10.4049/jimmunol.179.3.1449

273 Hayes, K. S., Bancroft, A. J. & Grencis, R. K. The role of TNF-alpha in Trichuris muris infection II: global enhancement of ongoing Th1 or Th2 responses. *Parasite Immunol* **29**, 583-594 (2007). https://doi.org:10.1111/j.1365-3024.2007.00980.x

274 Choi, J. P. *et al.* TNF-alpha is a key mediator in the development of Th2 cell response to inhaled allergens induced by a viral PAMP double-stranded RNA. *Allergy* **67**, 1138-1148 (2012). https://doi.org:10.1111/j.1398-9995.2012.02871.x

275 Lee, H. S. *et al.* TNF-alpha enhance Th2 and Th17 immune responses regulating by IL23 during sensitization in asthma model. *Cytokine* **79**, 23-30 (2016). https://doi.org:10.1016/j.cyto.2015.12.001

276 Torrente, Y. *et al.* Tumor necrosis factor-alpha (TNF-alpha) stimulates chemotactic response in mouse myogenic cells. *Cell Transplant* **12**, 91-100 (2003). https://doi.org:10.3727/000000003783985115

277 Lane, B. R. *et al.* TNF-alpha inhibits HIV-1 replication in peripheral blood monocytes and alveolar macrophages by inducing the production of RANTES and decreasing C-C chemokine receptor 5 (CCR5) expression. *J Immunol* **163**, 3653-3661 (1999).

278 Ohnesorge, N. *et al.* Erk5 activation elicits a vasoprotective endothelial phenotype via induction of Kruppel-like factor 4 (KLF4). *J Biol Chem* **285**, 26199-26210 (2010). https://doi.org:10.1074/jbc.M110.103127

279 Hurwitz, A. A., Lyman, W. D. & Berman, J. W. Tumor necrosis factor alpha and transforming growth factor beta upregulate astrocyte expression of monocyte chemoattractant protein-1. *J Neuroimmunol* **57**, 193-198 (1995). https://doi.org:10.1016/0165-5728(95)00011-p

280 Ozaki, K. *et al.* Interleukin-1 beta and tumor necrosis factor-alpha stimulate synergistically the expression of monocyte chemoattractant protein-1 in fibroblastic cells derived from human periodontal ligament. *Oral Microbiol Immunol* **11**, 109-114 (1996). https://doi.org:10.1111/j.1399-302x.1996.tb00344.x

281 Brzezinska-Blaszczyk, E., Pietrzak, A. & Misiak-Tloczek, A. H. Tumor necrosis factor (TNF) is a potent rat mast cell chemoattractant. *J Interferon Cytokine Res* **27**, 911-919 (2007). https://doi.org:10.1089/jir.2006.0158

282 Sugita, S. *et al.* Inhibition of Th17 differentiation by anti-TNF-alpha therapy in uveitis patients with Behcet's disease. *Arthritis Res Ther* **14**, R99 (2012). https://doi.org:10.1186/ar3824

283 Zhang, Q. *et al.* TNF-alpha impairs differentiation and function of TGF-beta-induced Treg cells in autoimmune diseases through Akt and Smad3 signaling pathway. *J Mol Cell Biol* **5**, 85-98 (2013). https://doi.org:10.1093/jmcb/mjs063

284 Biton, J. *et al.* Interplay between TNF and regulatory T cells in a TNF-driven murine model of arthritis. *J Immunol* **186**, 3899-3910 (2011). https://doi.org:10.4049/jimmunol.1003372

285 Baba, N., Rubio, M. & Sarfati, M. Interplay between CD45RA+ regulatory T cells and TNF-alpha in the regulation of human Th17 differentiation. *Int Immunol* **22**, 237-244 (2010). https://doi.org:10.1093/intimm/dxp130

286 Wu, L. Y., Li, X., Chang, C. K., Ying, S. X. & Pu, Q. [Abnormal polarization of T lymphocyte induces negative hematopoietic regulation in myelodysplastic syndromes]. *Zhonghua Xue Ye Xue Za Zhi* **28**, 549-554 (2007).

287 Majka, M. *et al.* Numerous growth factors, cytokines, and chemokines are secreted by human CD34(+) cells, myeloblasts, erythroblasts, and megakaryoblasts and regulate normal hematopoiesis in an autocrine/paracrine manner. *Blood* **97**, 3075-3085 (2001). https://doi.org:10.1182/blood.v97.10.3075

288 Skobin, V., Jelkmann, W., Morschakova, E., Pavlov, A. D. & Schlenke, P. Tumor necrosis factor-alpha and TNF-beta inhibit clonogenicity of mobilized human hematopoietic progenitors. *J Interferon Cytokine Res* **20**, 507-510 (2000). https://doi.org:10.1089/10799900050023924

289 Neurath, M. F., Finotto, S. & Glimcher, L. H. The role of Th1/Th2 polarization in mucosal immunity. *Nat Med* **8**, 567-573 (2002). https://doi.org:10.1038/nm0602-567

290 Lin, H. *et al.* Total glucosides of paeony ameliorates TNBS-induced colitis by modulating differentiation of Th17/Treg cells and the secretion of cytokines. *Mol Med Rep* **16**, 8265-8276 (2017). https://doi.org:10.3892/mmr.2017.7598

291 Akoum, A., Lemay, A., Brunet, C. & Hebert, J. Secretion of monocyte chemotactic protein-1 by cytokine-stimulated endometrial cells of women with endometriosis. Le groupe d'investigation en gynecologie. *Fertil Steril* **63**, 322-328 (1995). https://doi.org:10.1016/s0015-0282(16)57363-4

292 Buhrmann, C. *et al.* Induction of the Epithelial-to-Mesenchymal Transition of Human Colorectal Cancer by Human TNF-beta (Lymphotoxin) and its Reversal by Resveratrol. *Nutrients* **11** (2019). https://doi.org:10.3390/nu11030704

293 Zoja, C. *et al.* Interleukin-1 beta and tumor necrosis factor-alpha induce gene expression and production of leukocyte chemotactic factors, colony-stimulating factors, and interleukin-6 in human mesangial cells. *Am J Pathol* **138**, 991-1003 (1991).

294 Kownatzki, E., Kapp, A. & Uhrich, S. Modulation of human neutrophilic granulocyte functions by recombinant human tumor necrosis factor and recombinant human lymphotoxin. Promotion of adherence, inhibition of chemotactic migration and superoxide anion release from adherent cells. *Clin Exp Immunol* **74**, 143-148 (1988).

295 Czepluch, F. S., Olieslagers, S., van Hulten, R., Voo, S. A. & Waltenberger, J. VEGF-A-induced chemotaxis of CD16+ monocytes is decreased secondary to lower VEGFR-1 expression. *Atherosclerosis* **215**, 331-338 (2011). https://doi.org:10.1016/j.atherosclerosis.2011.01.004

296 Waltenberger, J., Lange, J. & Kranz, A. Vascular endothelial growth factor-A-induced chemotaxis of monocytes is attenuated in patients with diabetes mellitus: A potential predictor for the individual capacity to develop collaterals. *Circulation* **102**, 185-190 (2000). https://doi.org:10.1161/01.cir.102.2.185

297 Ryu, J. K., Cho, T., Choi, H. B., Wang, Y. T. & McLarnon, J. G. Microglial VEGF receptor response is an integral chemotactic component in Alzheimer's disease pathology. *J Neurosci* **29**, 3-13 (2009). https://doi.org:10.1523/JNEUROSCI.2888-08.2009

298 Henriksen, K., Karsdal, M., Delaisse, J. M. & Engsig, M. T. RANKL and vascular endothelial growth factor (VEGF) induce osteoclast chemotaxis through an ERK1/2-dependent

mechanism. *J Biol Chem* **278**, 48745-48753 (2003). https://doi.org:10.1074/jbc.M309193200
299 Ancelin, M. *et al.* Vascular endothelial growth factor VEGF189 induces human neutrophil chemotaxis in extravascular tissue via an autocrine amplification mechanism. *Lab Invest* **84**, 502-512 (2004). https://doi.org:10.1038/labinvest.3700053
300 de Paulis, A. *et al.* Expression and functions of the vascular endothelial growth factors and their receptors in human basophils. *J Immunol* **177**, 7322-7331 (2006). https://doi.org:10.4049/jimmunol.177.10.7322
301 Hirsch, L., Flippot, R., Escudier, B. & Albiges, L. Immunomodulatory Roles of VEGF Pathway Inhibitors in Renal Cell Carcinoma. *Drugs* **80**, 1169-1181 (2020). https://doi.org:10.1007/s40265-020-01327-7
302 Mor, F., Quintana, F. J. & Cohen, I. R. Angiogenesis-inflammation cross-talk: vascular endothelial growth factor is secreted by activated T cells and induces Th1 polarization. *J Immunol* **172**, 4618-4623 (2004). https://doi.org:10.4049/jimmunol.172.7.4618
303 Kim, Y. S. *et al.* Vascular endothelial growth factor is a key mediator in the development of T cell priming and its polarization to type 1 and type 17 T helper cells in the airways. *J Immunol* **183**, 5113-5120 (2009). https://doi.org:10.4049/jimmunol.0901566
304 Grotz, T. E. *et al.* Evidence of Th2 polarization of the sentinel lymph node (SLN) in melanoma. *Oncoimmunology* **4**, e1026504 (2015). https://doi.org:10.1080/2162402X.2015.1026504
305 Nevala, W. K. *et al.* Evidence of systemic Th2-driven chronic inflammation in patients with metastatic melanoma. *Clin Cancer Res* **15**, 1931-1939 (2009). https://doi.org:10.1158/1078-0432.CCR-08-1980
306 Boissel, N. *et al.* Defective blood dendritic cells in chronic myeloid leukemia correlate with high plasmatic VEGF and are not normalized by imatinib mesylate. *Leukemia* **18**, 1656-1661 (2004). https://doi.org:10.1038/sj.leu.2403474
307 Kim, Y. S. *et al.* Distinct roles of vascular endothelial growth factor receptor-1- and receptor-2-mediated signaling in T cell priming and Th17 polarization to lipopolysaccharide-containing allergens in the lung. *J Immunol* **185**, 5648-5655 (2010). https://doi.org:10.4049/jimmunol.1001713
308 Terme, M. *et al.* VEGFA-VEGFR pathway blockade inhibits tumor-induced regulatory T-cell proliferation in colorectal cancer. *Cancer Res* **73**, 539-549 (2013). https://doi.org:10.1158/0008-5472.CAN-12-2325
309 Purpura, K. A. *et al.* Soluble Flt-1 regulates Flk-1 activation to control hematopoietic and endothelial development in an oxygen-responsive manner. *Stem Cells* **26**, 2832-2842 (2008). https://doi.org:10.1634/stemcells.2008-0237
310 Martin, R. *et al.* SCL interacts with VEGF to suppress apoptosis at the onset of hematopoiesis. *Development* **131**, 693-702 (2004). https://doi.org:10.1242/dev.00968
311 Fu, Z. *et al.* Curcumin inhibits angiogenesis and improves defective hematopoiesis induced by tumor-derived VEGF in tumor model through modulating VEGF-VEGFR2 signaling pathway. *Oncotarget* **6**, 19469-19482 (2015). https://doi.org:10.18632/oncotarget.3625
